# Magnetron Sputtering of Pure δ-Ni_5_Ga_3_ Thin Films for CO_2_ Hydrogenation

**DOI:** 10.1021/acscatal.4c03345

**Published:** 2024-08-06

**Authors:** Filippo Romeggio, Jonathan F. Schouenborg, Peter C. K. Vesborg, Ole Hansen, Jakob Kibsgaard, Ib Chorkendorff, Christian D. Damsgaard

**Affiliations:** † DTU Physics, Technical University of Denmark, Kongens Lyngby DK-2800, Denmark; ‡ DTU Nanolab, Technical University of Denmark, Kongens Lyngby DK-2800, Denmark

**Keywords:** thermocatalysis, CO_2_ hydrogenation, methanol, δ-Ni_5_Ga_3_, thin films, magnetron sputtering

## Abstract

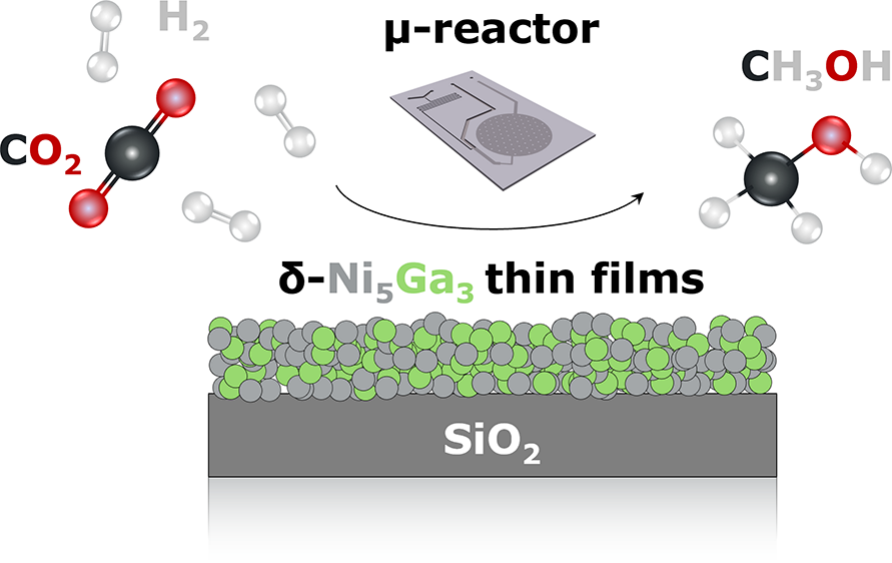

Previous studies have identified δ-Ni_5_Ga_3_ as a promising catalyst for the hydrogenation of CO_2_ to methanol at atmospheric pressure. Given its recent discovery, the current understanding of this catalyst is very limited. Additionally, the presence of multiple thermodynamically stable crystal phases in the Ni/Ga system complicates the experiments and their interpretation. Conventional synthesis methods often result in the production of unwanted phases, potentially leading to incorrect conclusions. To address this issue, this study focuses on the synthesis of pure δ-Ni_5_Ga_3_ using magnetron sputtering deposition followed by low-temperature H_2_ annealing. Extensive characterization confirmed the reproducible synthesis of well-defined δ-Ni_5_Ga_3_ thin films. These films, deposited directly into state-of-the-art μ-reactors, demonstrated methanol production at low temperatures and maintained a high stability over time. This method allowed for detailed surface and bulk characterization before and after the reaction, providing a comprehensive understanding of the deactivation mechanism. Our findings significantly contribute to the understanding of the Ni/Ga system and its behavior during catalytic activity, deactivation, and regeneration. This study also sets an example of how physical synthesis methods such as magnetron sputtering can be effectively employed to investigate complex catalytic systems, offering a viable alternative to more elaborate chemical methods.

## 1 Introduction

One of the strategies to reduce CO_2_ is to convert it to valuable carbon-based products. Methanol, with its large-scale demand (over 111 Mt/yr in 2022) and versatility as a feedstock chemical, is an attractive target product.^1^

Methanol can be easily produced using CO_2_ obtained from single-point capture or direct air capture. Ideally, this process utilizes hydrogen generated through the electrolysis of water powered by renewable energy sources. The growing demand to reduce CO_2_ emissions and comply with antiflaring regulations is driving the methanol market toward small-scale plants located near the end-users. Typically, when renewable feedstocks such as CO_2_ waste streams and electrolysis are used, one or more components of the synthesis gas are supplied at a low pressure. This is particularly true when dealing with pure CO_2_ feedstock, as opposed to the traditional CO/CO_2_ mixtures used in industrial methanol synthesis. Using pure CO_2_ causes the formation of CO as a byproduct through the reverse water–gas shift (RWGS) reaction. In conventional methanol production, operational pressures are generally maintained between 20 and 50 bar to balance the thermodynamics of the CO_2_ hydrogenation reaction and to enhance the reaction rates.^2^ However, operating at such high pressures involves significant costs related to the reactor design, material requirements, and energy consumption. Therefore, this study explores the feasibility of performing CO_2_ hydrogenation at a much lower pressure of 1 bar. Operating at low pressure also allows investigation of the fundamental aspects of the reaction kinetics and catalyst behavior in a simplified environment. This approach bridges the pressure gap between high-pressure industrial methanol synthesis and ultrahigh vacuum (UHV) characterization techniques. This enables the progressive optimization and adaptation of findings for higher pressure applications, potentially facilitating a smoother transition from laboratory-scale experiments to industrial-scale processes.

Bimetallic catalysts are promising candidates for CO_2_ hydrogenation because they allow fine-tuning of the catalytic properties by modifying the active sites and adjacent atoms, resulting in different binding strengths of reactive intermediates. For example, copper alone is not a good catalyst for CO_2_ hydrogenation to methanol, but the bimetallic nature of the Cu–Zn sites in the commercial Cu/ZnO/Al_2_O_3_ catalyst results in high activities for CO/CO_2_ hydrogenation to methanol.^3^ Other bimetallic catalysts that leverage this synergy include late transition metals of the 10th and 11th groups, mixed with reducible atoms such as In, Zn, and Ga.^4^

The Ni/Ga system has attracted a great deal of attention in the catalysis community after its discovery in 2014.^5^ It was shown both theoretically and experimentally that some alloys made from these metals have the potential to perform as the commercial CuZn catalyst, with improved stability against sintering.^5^ δ-Ni_5_Ga_3_ was found to be the most promising Ni/Ga alloy configuration. To date, only around 30 papers have been published on the use of this catalyst for CO_2_ hydrogenation (approximately 80% of which were published in the last 5 years). In an attempt to synthesize the desired phase (the Ni/Ga system counts multiple thermodynamically stable phases, as shown in Figure S1), various experimental techniques have been used in the literature, most of which rely on incipient wetness impregnation and coprecipitation. The first reported synthesis of the catalyst involved the reduction of nickel and gallium nitrates at 700 °C in H_2_ for 2 h.^5^ More studies followed the same procedure,^6−8^ or used coprecipitation followed by the same treatment conditions.^9^ As shown in the in situ XRD experiments of Sharafutdinov et al.,^6^ with this procedure, the final diffraction pattern still contains 15% of -Ni_3_Ga_1_. Attempts to synthesize the catalyst at a lower temperature with this technique would therefore result in a mix of Ni/Ga phases.^10−14^ Other groups attempted the synthesis by adding a precalcination step at ≥400 °C to remove nitrates,^15−21^ but reached similar results. Another popular technique combines a calcination step at 500 °C and treatment with NaBH_4_ in ethanol,^15,16,22−24^ with the idea of converting the mixed oxides of Ni^2+^ and Ga^3+^ to Ni–Ga alloys. Even in this case, when looking at the diffractograms, it is unclear whether a pure δ-Ni_5_Ga_3_ phase was obtained. A more unusual approach was done by Cuenya et al.,^25^ where samples were prepared using inverse micelle encapsulation, calcined at 470 °C for 6 h and a reduction at 700 °C in H_2_ for 7.5 h. Unfortunately, given the small particle size, it is difficult to interpret the XRD data. Moreover, also in this case, there seems to be a coexistence of -Ni_3_Ga_1_ and δ-Ni_5_Ga_3_. Lastly, two studies were published on the use of cografting,^26^ where the focus was on different ratios of Ni/Ga rather than on the crystallinity of the catalyst, and ball-milling,^27^ where a broad mix of Ni/Ga phases was present. An overview of all of the published papers on δ-Ni_5_Ga_3_ is available in Table S1. The presence of -Ni_3_Ga_1_ in all these catalysts means that some gallium could be present in an unalloyed state. The presence of two phases together with some unalloyed gallium makes the interpretation of these results extremely difficult.

Despite the system complexity and the struggle to synthesize the desired phase, researchers have investigated the catalytic performance of δ-Ni_5_Ga_3_ using various techniques, including in situ and operando spectroscopy. These studies provided valuable insights into the Ni/Ga system’s behavior during CO_2_ hydrogenation reactions. However, the totality of these studies attempted the synthesis of this alloy via chemical methods in the form of nanoparticles, inevitably introducing more variables that need to be considered when trying to understand the nature of the catalyst (such as particle size dependence, support influence, ligand impact, etc.).^28^ Because of the small loading and crystallite size, nanoparticle-based systems are not always suitable for characterization techniques such as XRD, especially when on flat supports. This often makes it difficult to prove that the synthesized material is the desired δ-Ni_5_Ga_3_, considering the similarities between the different phases. As a consequence, comparing the results across different studies and drawing definite conclusions about the catalyst’s performance have been challenging. Thus, the Ni/Ga system is still far from being fully understood. Investigating alternative synthesis methods could help solve some of these problems. For example, using thin films instead of nanoparticle-based catalysts reduces the variables to consider when analyzing their performances. With a uniform layer, the support becomes much less important and can be excluded from the discussions. Deactivation by sintering, one of the biggest problems with nanoparticle catalysis,^29^ can also be neglected—allowing to focus instead on other types of deactivation mechanisms. Magnetron sputtering appears as one of the top candidates for controlled thin film deposition,^30^ with the advantage of ensuring high purity of the deposited materials by avoiding the presence of ligands that would otherwise need to be removed (e.g., via UV light exposure, specific solvent washing treatments, and plasma treatments^31−34^ to prevent impacting the reaction.

**Figure 1:**
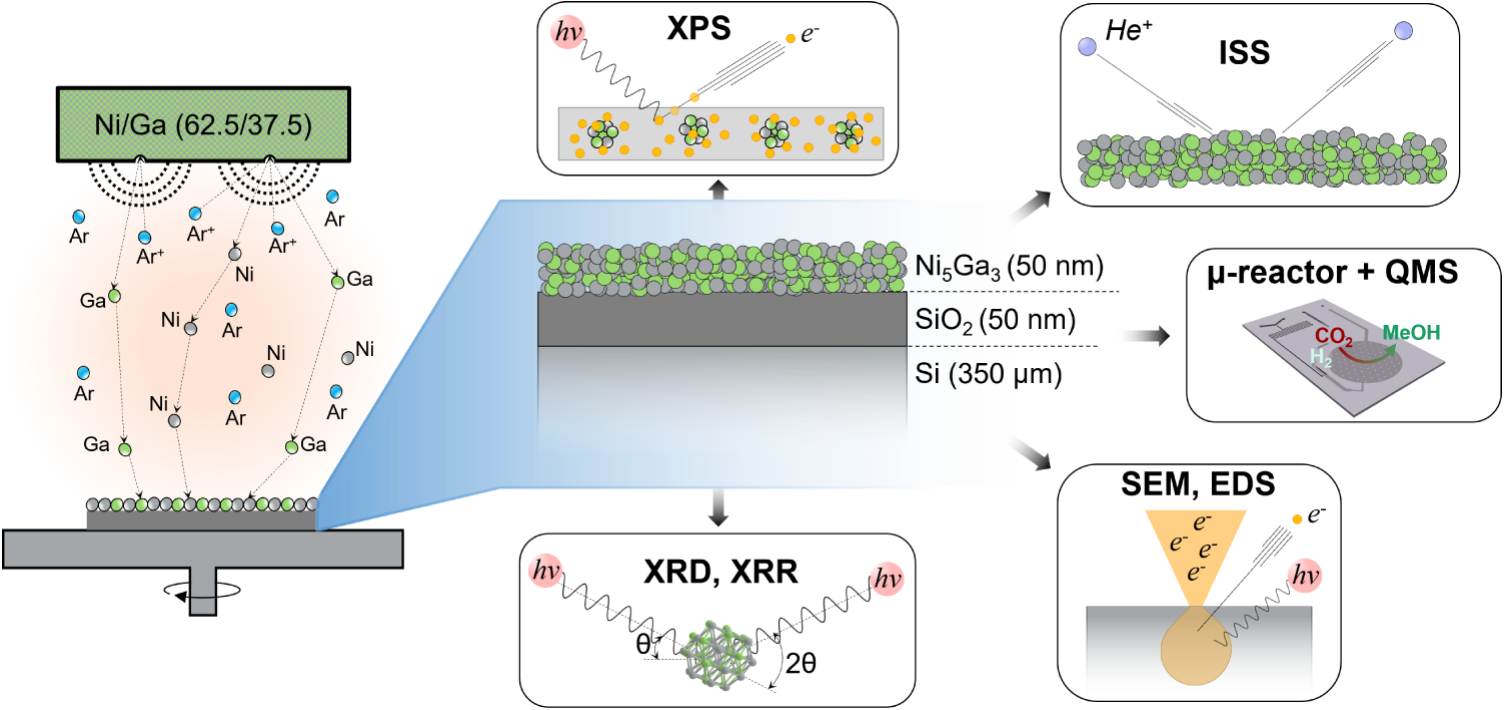
Overview of methods for catalyst characterization. 50 nm thin films of δ-Ni_5_Ga_3_ were produced via magnetron sputtering and their structure was studied using XRD, XRR, XPS, ISS, SEM, and EDS. The catalytic activity was investigated with μ-reactors coupled with a QMS.

In this article, well-defined thin films of δ-Ni_5_Ga_3_ are synthesized via magnetron sputtering. We present this technique as a promising alternative to the chemical methods reported in the literature for the synthesis of this catalyst, in particular with regards to phase purity, as well as suitability to characterization techniques. Thin films, compared to nanoparticles, also allow to exclude possible effects from the support, interfaces, and particle size on the catalytic activity, potentially helping to disentangle the complex nature of the catalyst. A similar synthesis was attempted by Shidong et al. in 2018,^35^ but with a focus on different phases of the Ni/Ga system, not active for CO_2_ hydrogenation to methanol. In this study, the activity and stability of the samples were tested in μ-reactors at temperatures between 25 °C and 385 °C at 1 bar. A combination of bulk and surface-sensitive techniques was utilized to investigate the catalyst at different stages, as summarized in Figure 1. This manuscript features three main achievements: first, a description of the synthesis of a well-defined δ crystal phase is reported with a different method and at unprecedented low temperatures; second, the catalyst is shown to be active toward methanol under milder conditions compared to the literature; third, a more complete picture of the deactivation mechanism is given.

## 2 Experimental Section

### 2.1 Catalyst Preparation

The thin films were deposited on state-of-the-art μ-reactors for thermal activity tests^36^ (Figures S2 and S3) and on rectangular dummy chips (5 mm × 15 mm, Figure S4) for characterization purposes following the same procedure reported in ref (37). Both surfaces consist of an Si single crystal with a 50 nm SiO_2_ layer on top, grown thermally. The substrates were wiped with ethanol and dried with a CO_2_ blower in order to remove surface contaminations (Figure S5). Immediately thereafter, they were mounted into a magnetron sputtering system with a base pressure in the low 10^–9^ mbar range. The samples were first cleaned with Ar plasma (30 W, 4 × 10^–3^ mbar, 2 min). Afterward, a 50 nm layer of δ-Ni_5_Ga_3_ was deposited using DC sputtering of a bimetallic target (Ni/Ga = 62.5/37.5 At %), by applying 20 W. The deposition rate (0.35 Å/s) was calibrated using a Quartz Crystal Microbalance at a density of 9.1 g/cm^3^. A constant pressure of 4 × 10^–3^ mbar was maintained, and the sample was rotated throughout the whole deposition to ensure a uniform coverage. The thickness was confirmed with XRR and cross-sectional SEM images.

### 2.2 CO_2_ Hydrogenation Measurements

We conducted activity measurements using state-of-the-art equipment. The system we used had a total reaction volume of approximately 240 nL, and the gas flows involved are on the order of nmol/min. One notable feature of this system is that all of the gases entering the reactive area flow directly to the mass spectrometer without any dilution or carrier gas, enabling highly sensitive detection of reaction products. On the back side of the setup, a 50 nm platinum thin film is employed to control and measure the temperature using resistive heating and a resistance temperature detector. Temperature measurements were also conducted using a thermocouple on the Pyrex lid area situated above the reactive surface. To seal the μ-reactors, we utilized anodic bonding technique (see Section S3.4). The catalysts were exposed to a constant inlet flow of around 35 nmol/min with a composition of CO_2_:H_2_:Ar = 1:3:0.5, where argon (Ar) was used as a control gas. The CO_2_:H_2_ ratio of 1:3 was found to be the optimum for methanol activity (Figure S6). Temperature ramps were performed at a rate of 4 °C per minute, reaching temperatures of up to 385 °C. These ramps were carried out to assess the relative activity and stability of the catalysts under different conditions, with each step lasting 1 h after every 15 °C increment. The products were analyzed using a Pfeiffer vacuum QMG 422 quadrupole mass spectrometer (QMS). The QMS signal was calibrated using a baratron (Section S5) to determine the product flow rate in moles per second. All of the lines connecting the μ-reactor outlet to the QMS were heated to temperatures exceeding 100 °C to prevent condensation of methanol and water.

### 2.3 X-Ray Photoelectron Spectroscopy and Ion Scattering Spectroscopy

After the catalyst was synthesized in the magnetron sputtering chamber, high vacuum conditions were broken, and the samples were immediately loaded into a Theta Probe X-ray photoelectron spectrometer from Thermo Scientific. The surface composition was measured by X-ray photoelectron spectroscopy (XPS) using a monochromatic Al Kα X-ray source (1486.68 eV). The XPS spot size was set to 400 μm, the pass energy was set to 50 eV, and the step size was set to 0.1 eV. For each core level, 50 scans were averaged. The data analysis and peak fitting were performed using the Avantage software. The survey spectrum and details of the Ni and Ga peak fittings are given in Figure S7 and Section S4.1.1. Ion scattering spectroscopy (ISS) was performed with He^+^ ions, using an acceleration voltage of 1 keV, an emission current of 1 mA, and an He pressure of 1 × 10^–7^ mbar (see more details in Section S4.1.2 and Figure S8).

### 2.4 Scanning Electron Microscopy

The surface of the μ-reactors was studied by scanning electron microscopy (SEM) using the Thermo Scientific Helios 5 Hydra UX PFIB microscope operating at an acceleration voltage of 5 kV, a current of 0.20 nA, and at a height of 4 mm, and using a secondary electron through the lens detector (TLD) in immersion mode.

### 2.5 Energy Dispersive X-Ray Spectroscopy

Energy dispersive X-ray spectroscopy (EDS/EDX) measurements were taken with a Quanta FEG 250 Analytical ESEM equipped with an X-Max50 detector at an acceleration voltage of 30 kV, a spot size of 5.5, and a working distance of 10 mm. The chamber was maintained at a pressure of approximately 5 × 10^–6^ mbar. For more information about measurements and quantification, see Section S4.1.3 and Figure S9.

### 2.6 Grazing-Incidence X-Ray Diffraction and X-Ray Reflectivity

Grazing-incidence X-ray diffraction (GI-XRD) and X-ray reflectivity (XRR) were performed using a Panalytical Empyrean X-ray diffractometer operating at an acceleration voltage of 45 kV and a current of 40 mA with a Cu X-ray source. The incident optics used during the measurements were a 1/32 divergence slit, a parallel beam mirror for Cu X-rays, a 0.04 rad soller slit, and a 4 mm mask. A 0.1 mm Cu attenuator was used for the XRR measurements, together with a 2 mm mask. The reflected optics were a parallel plate collimator, a 0.04 rad soller slit, and a Panalytical PIXcel3D detector in open detector configuration. The XRD measurements were done at an incidence angle of 0.550 ω, whereas the reflectivity curves were recorded using a ω-2θ scan. The data were analyzed using the X’Pert Reflectivity software to determine the thickness and density of the deposited films (Section S4.1.3 and Figure S10).

## 3 Results and Discussion

### 3.1 Well-Defined δ-Ni_5_Ga_3_

**Figure 2:**
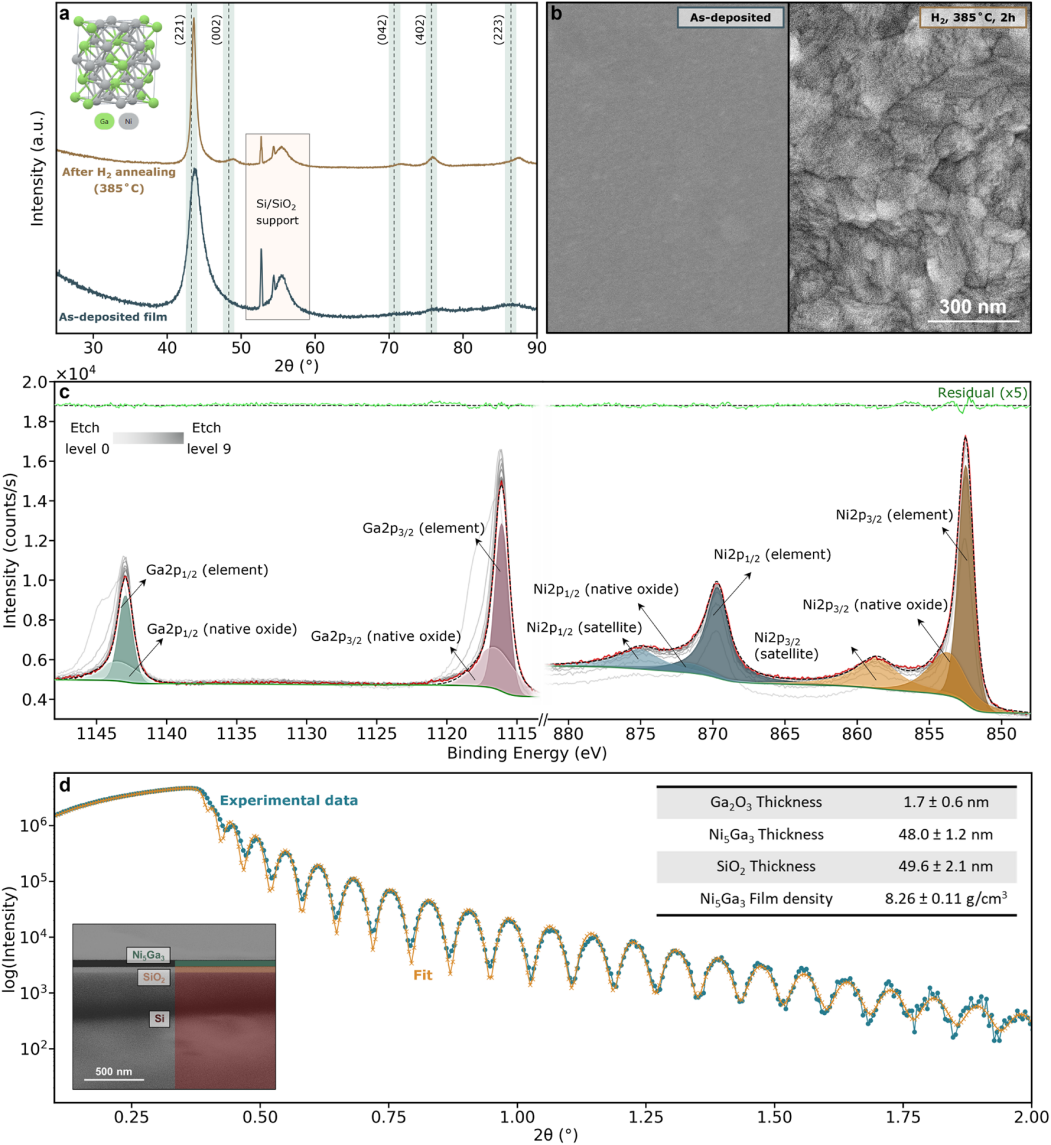
Characterization of the films before the reaction. a) XRD patterns and b) SEM images before and after thermal treatment in H_2_ at 385 °C. c) XPS fitting of Ga 2p and Ni 2p core levels on an as-deposited sample. d) XRR fitting of the as-deposited δ-Ni_5_Ga_3_ thin films and the resulting film thicknesses. The inset at the bottom left is a cross-sectional image of one sample. The XRD patterns were normalized to the maximum intensity of the (221) peak. The δ-Ni_5_Ga_3_ reference pattern was taken from ICSD (Coll. Code 103861).^38^

Right after deposition via magnetron sputtering, the thin films were analyzed by GI-XRD to check their bulk crystallinity. The broad peaks observed in Figure 2a indicate that it was possible to synthesize small crystal grains of the desired δ-Ni_5_Ga_3_ at room temperature. Given that a multitude of peaks are shared by different phases of Ni/Ga in that range, the broad peaks inhibit the unambiguous proof of pure δ-Ni_5_Ga_3_ synthesis. Subsequent annealing of the catalyst in H_2_ for 2 h at 385 °C was therefore performed, which resulted in substantial crystal growth, as evident from both XRD and SEM results (Figure 2a,b). Figure S11 reports the same XRD data but without normalization to help visualize the crystal and phase growth. Performing the same treatment in the absence of H_2_ would not lead to the same crystal growth, as seen in Figure S12. The catalyst diffractogram shows peak positions that are comparable to the δ-Ni_5_Ga_3_ reference,^38^ with minor peak shifts attributed to stress in the thin film. This demonstrates the feasibility of synthesizing the right phase of δ-Ni_5_Ga_3_ at the unprecedented temperature of 385 °C. Other than relying on lower temperatures compared to the literature, the synthesis has the advantage of being a clean, reproducible, and ligand-free technique.

The atomic composition of the films’ surface was measured using XPS and ISS, as can be seen in Figures 2c and S8, respectively. First, the surface was analyzed with XPS in order to check for any eventual contaminations. The samples were exposed to air before being loaded into the UHV chamber for the measurements. Therefore, it was necessary to perform some Ar^+^ etching cycles to remove the deposited adventitious carbon and the surface-oxidized Ga (lines shown in gray in Figure 2c). XPS fitting of Ga 2p and Ni 2p core levels in Figure 2c revealed an atomic percentage of Ni/Ga of 65/35, in good agreement with the theoretical 67.5/32.5 At% ratio for δ-Ni_5_Ga_3_. To have a better understanding of the surface composition and to exclude contaminations, ISS was also performed. The results in Figure S8 show a peak appearing at 784 eV after some etching cycles, which was attributed to Ga. After some more etching cycles, one more peak appears at 758 eV (assigned to Ni). The ISS results confirm that the surface, after air exposure, is contaminated with adventitious carbon and presents a few monolayers of oxidized gallium. Since in both the XPS survey scan (Figure S7) and the ISS no unidentified peaks were present, the surface was considered to be entirely composed of nickel, gallium, and oxygen, with possible surface contaminations below the detection limit of ISS. The catalyst bulk atomic composition, analyzed via EDS, also reflected the expected Ni to Ga ratio (Figure S9). Despite all the characterization techniques point to the conclusion that the sample has an Ni/Ga ratio of 5/3, it has to be made clear that the sample surface during reaction conditions could look slightly different. As an example, Gallo et al. reported the catalyst activity toward methanol to be correlated with the presence of Ga_2_O_3_ on the surface,^7^ in a similar fashion to the Pd/Ga_2_O_3_ and Cu/Ga_2_O_3_ synergy.^39−45^

To confirm the uniformity across various depositions, multiple SEM images were taken across the whole samples areas. XRR was also performed on the as-deposited sample (before H_2_ annealing). The curve fitting allowed us to obtain information like film density and thickness. Figure 2d shows the summarized data obtained from the XRR measurements (see Section S4.1.4 for more information). The resulting film density of 8.26 ± 0.11 g/cm^3^ matched very well with the theoretical bulk density of δ-Ni_5_Ga_3_ (9.09 g/cm^3^), with slightly lower values, most probably because of small voids in the films (which are intrinsic to the synthesis method). The thicknesses of the films were repeatably measured to be around 50 nm (both for the SiO_2_ substrate and the deposited δ-Ni_5_Ga_3_). The SEM inset also visually confirms the uniform coverage and the thickness of the layers.

Our synthesis approach stands out when compared to the existing literature (Table S1): as broadly discussed in the Introduction, previously reported methods for δ-Ni_5_Ga_3_ synthesis require harsh conditions such as hydrogenation at 600/700 °C for 7 h^10^ or 500 °C calcination combined with NaBH_4_/EtOH chemical treatment.^15,16,22−24^ In contrast, our method allowed for a clean and reproducible synthesis of a pure δ-Ni_5_Ga_3_ phase through magnetron sputtering followed by ex situ H_2_ annealing, at an unprecedented temperature of 385 °C, without the need for any chemical treatment.

This work highlights the efficacy of physical techniques for synthesizing model catalysts. The ability to obtain pure crystal phases under such mild conditions could be extended to a multitude of alloy materials, opening new avenues for catalytic research.

### 3.2 Catalytic Performance

**Figure 3:**
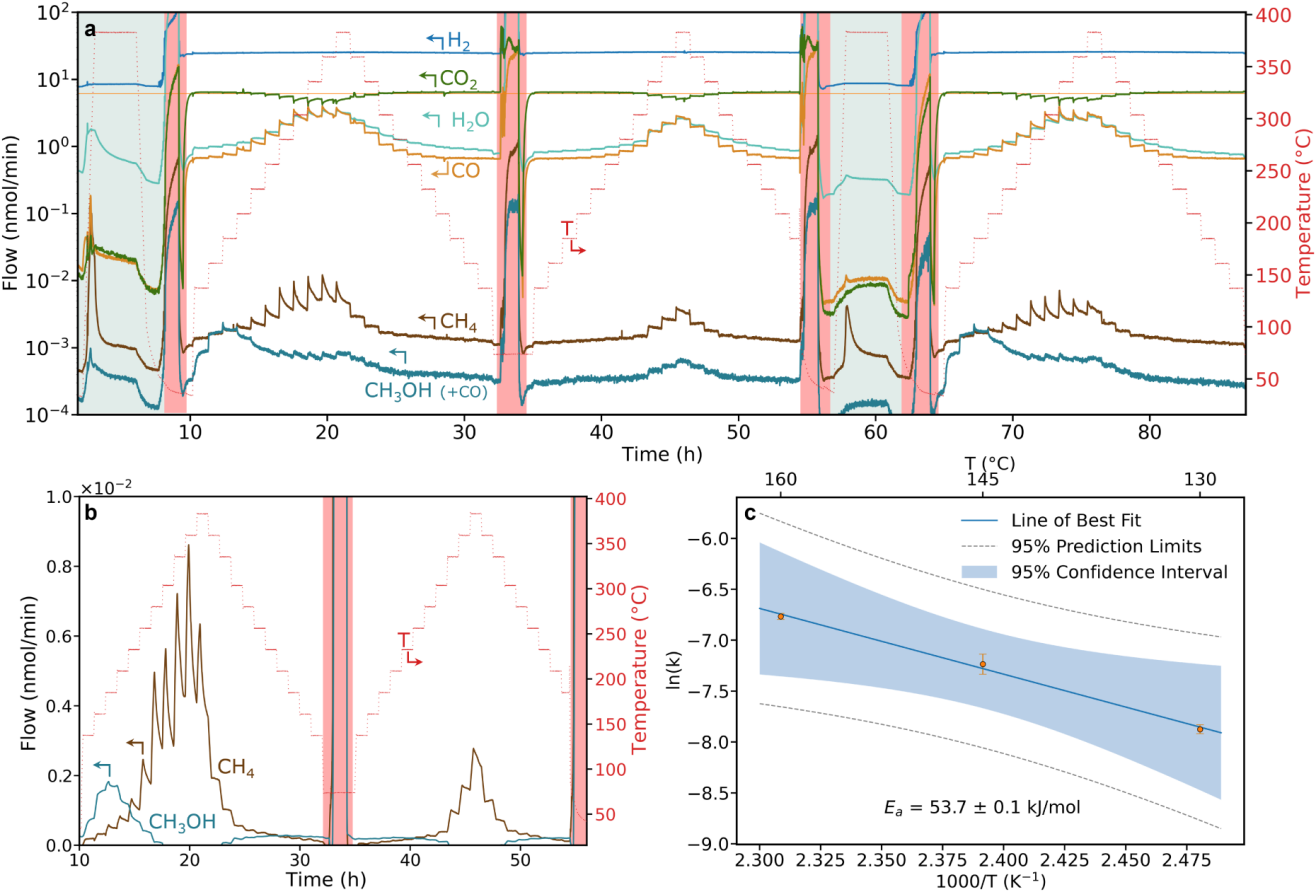
δ-Ni_5_Ga_3_ catalytic performance: a) typical catalytic activity testing of 50 nm sputter-deposited thin film under CO_2_ hydrogenation conditions (1 bar, H_2_:CO_2_:Ar = 3:1:0.5). Green areas represent activation of the catalyst (1 bar, H_2_:Ar = 2:0.5), whereas red areas indicate reactor pump-downs and are therefore to be disregarded. The different gases were detected in the QMS by scanning for the following masses: H_2_ = M2, CO_2_ = M44, H_2_O = M18, CH_4_ = M15, and CH_3_OH = M31. The flows were calculated from a raw QMS signal which was calibrated for all the different masses, but not further treated to avoid misinterpretation. Therefore, M31 results as a combination of CH_3_OH and ^13^C^18^O; hence, the M31 signal at high temperatures. For more information, see Section 4.1.2. b) Baseline- and isotope-corrected catalytic activity testing of CH_3_OH and CH_4_ to highlight the catalyst selectivity. In this plot, the CO signal that contributes to M31 is suppressed. The baseline-corrected full plot is reported in Figure S13. c) The Arrhenius plot and calculated apparent activation energy. All of the experiments were conducted at 1 bar with Ar used as a reference gas (and therefore not shown in the plot).

All catalytic activity tests were performed using high product-sensitivity μ-reactors mounted in a low-pressure containment volume to avoid diffusion of atmospheric contamination into the reactor.^36,46^Figure 3a shows an example of a typical experiment: preactivation in H_2_, two temperature ramps, a reactivation in H_2_, and one last temperature ramp. The data were treated simply by multiplying the raw signals with sensitivity factors from the QMS calibration (Section S4.1.1). In order to make it easier to visualize the catalyst selectivity, Figures 3b and S13 show instead the methanol and methane signals after correcting the data by subtracting their baselines, cracking patterns, and isotopes.

At first, all the samples were pretreated in hydrogen at 385 °C in order to reduce the surface Ga_2_O_3_ and remove possible adventitious carbon deposited when exposed to the atmosphere. This step is necessary to incorporate metallic gallium into the Ni/Ga matrix, enabling the growth of δ-Ni_5_Ga_3_ crystallites, as demonstrated in the previous section. Avoiding this step or performing it at lower temperatures would result in poorer catalytic activity (Figure S14), most probably because of an incomplete reduction of the surface Ga_2_O_3_. Afterward, the catalysts were exposed to CO_2_ hydrogenation conditions to check their activity toward methanol. Temperature ramps of up to 385 °C were used to test the activity and deactivation of the system under harsh conditions. These tests were repeated ∼15 times for reproducibility purposes and to tackle specific questions on the catalyst behavior (activity, stability, deactivation, regeneration, etc.). The experiments displayed high reproducibility between different samples, even when synthesized around one year apart. Additional catalytic activity plots and information can be found in Section S4.3.

In the first temperature ramp of Figure 3a,b methanol starts being detectable at a temperature of 135 °C (or as low as 115 °C in a slower temperature ramp experiment—see Figure S15). It is important to note that although some of the products’ signals did not stabilize before the switching temperature, methanol’s and methane’s signals do not significantly change with extended waiting times. As illustrated in Figure S15, reducing the temperature ramp speed (with steps of 15 °C instead of 25 °C) fully mitigates this effect. Long time-on-stream experiments (see Section S4.4) further show that the system reaches 80% of the steady-state signal after 1 h, starting from no methanol production. Given the minimal impact of stabilization time, it is reasonable to utilize the results from the extended experiment in Figure 3a for analyzing and interpreting the catalyst behavior. Previous literature reports on the performance of δ-Ni_5_Ga_3_ at 1 bar show methanol production only at *T* > 160 °C, with a maximum turnover frequency (TOF) at 210/220 °C.^5,6,25^ In this study, the catalyst displays a superior catalytic activity at low temperatures, presenting a maximum at around 185/200 °C. The selectivity remains close to 90% until the same temperature, after which it quickly shifts toward CH_4_ (Figure 3b). The different behavior compared to the reports in the literature could originate from the new synthesis method implemented in this investigation. Synthesis via physical methods is characterized by the absolute absence of chemical precursors, making it a cleaner option compared to chemical methods. The absence of ligands on the surface might have a drastic influence on the catalytic activity, especially at low temperatures, when the turnover frequencies are the lowest. The synthesis also has an important influence on the catalyst bulk crystallinity (e.g., stress phenomena observed in Figure 2a) and on the grain size, potentially causing different crystal facets to be exposed to the reaction environment and therefore, modifying the catalyst activity profile. All of these considerations could be at the origin of the differences observed in this paper compared to the literature. From the Arrhenius plot of Figure 3c, the catalyst shows an apparent activation energy of around 53 kJ/mol, which is comparable to the commercial CuZn catalyst but slightly higher compared to the one reported in other studies on the same catalyst.^14^ It is important to highlight that the comparison of these numbers is mostly reliable when the samples are tested under the exact same conditions. Nevertheless, this serves as a further indication of the remarkable performances and differences of these sputter-deposited δ-Ni_5_Ga_3_ thin films for CO_2_ hydrogenation to methanol.

### 3.3 Deactivation Mechanism

In Figure 3a, methanol is not anymore detected on the first temperature ramp-down after having reached 385 °C. The ramp (between 35 and 55 h) suggests that the surface of the catalyst is fully deactivated, since no methanol is produced even at the lower temperature region. The methanol signal that appears at temperatures between 300 °C and 385 °C should be disregarded, since it was assigned to the CO isotope of mass 31 (with C^13^ and O^18^, as explained in Section S4.3.1 and Figure S16).

**Figure 4:**
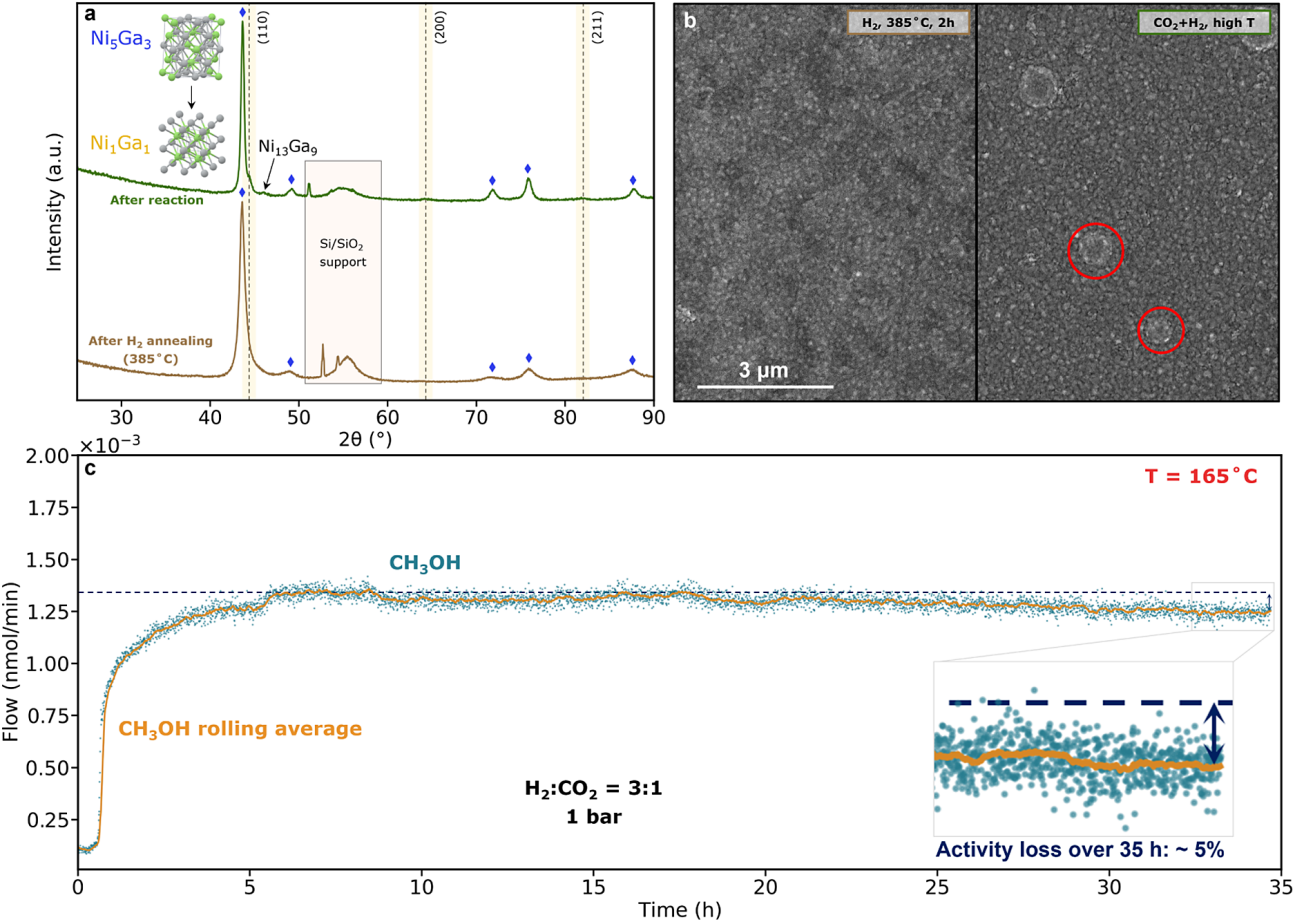
Deactivation mechanism. a) GI-XRD and b) SEM comparison pre- and postreaction (deactivated sample). The XRD references for Ni_1_Ga_1_ and Ni_13_Ga_9_ were taken from the simulated pattern in Materials Project (mp-1941 and mp-21589, respectively).^47^ The red circles in the SEM image indicate particles that appear only on the deactivated samples. c) Stability run over 35 h. The orange line was plotted as a rolling average of 20 data points.

To understand more about the deactivation mechanism of the catalyst, the experiment was stopped when the sample was fully deactivated (i.e., the condition of the catalyst at approximately 46 h in Figure 3a), and studied with GI-XRD and SEM. In the diffractogram of Figure 4a, it is possible to notice some crystalline features attributable to the Ni_1_Ga_1_ phase. Since this phase is not thermodynamically stable (energy above hull 0.043 eV/atom, see Figure S1), it is predicted to decompose to Ni_13_Ga_9_ and Ni_2_Ga_3_ (with a ratio close to 1:1).^47^ In the XRD plot, Ni_13_Ga_9_ is indeed noticeable together with Ni_1_Ga_1_ at 2θ degrees: 44.3, 46.0, 64.2, and 82.1. The presence of these on the surface is expected to inhibit methanol production, as only the 5/3 ratio was predicted active in the first paper from Studt et al.^5^ This is indeed what was observed in the second ramp of the current experiments, where only RWGS and CH_4_ production are observable, and no methanol is produced. Since 50% Ni_13_Ga_9_ + 50% Ni_2_Ga_3_ corresponds to a calculated Ni/Ga average ratio of 1.07, the ratio of Ni/Ga on the surface of the deactivated sample does not match the ratio of 1.67 of the as-synthesized compound. Therefore, the excess nickel is expected to aggregate in small crystallites (since not XRD detectable) or in amorphous islands. The postdeactivation samples observed through HR-SEM seem to confirm the previous hypothesis, showing round features homogeneously distributed on the surface (Figures 4b and S17). Characterization of these particles was attempted through EDS, GI-XRD, and XPS; but since in the probed area, the overall Ni/Ga average ratio would still be as the as-deposited 5/3, it is hardly possible to fully prove the nature of those particles. Nevertheless, it is likely that those features are attributable to the hypothesized dealloyed Ni. The presence of nickel on the surface would also further explain the selectivity shift toward CH_4_. Nevertheless, more focused characterization would be necessary to confirm the assumption around the nature of these surface particles.

The third temperature ramp of Figure 3a was performed as an attempt to reactivate the just-deactivated catalyst. Surprisingly, the methanol activity was fully recovered and presented the same values as in the first ramp. In Figure S17, it is possible to observe that the round features disappear after catalyst reactivation in H_2_. This finding proves that even after being exposed to very harsh conditions (385 °C in CO_2_ and H_2_), δ-Ni_5_Ga_3_ thin films can be reactivated to their original state. Therefore, this deactivation mechanism seems to be reversible, allowing the right crystal phase to form again under H_2_ treatment conditions, in a similar fashion to the initial catalyst activation. It is important to notice that during the reactivation in H_2_, some CH_4_ is detected as a product of reaction. This confirms the observations from the manuscript by Studt et al.,^5^ where the deactivation cause was attributed entirely to carbon deposition on the surface, covering the active sites, and therefore, decreasing the activity toward methanol. To check for carbon deposition, the reaction was stopped after fully deactivating one of the samples, which was then imaged with SEM and sputtered with a focused ion beam. In Figure S18, it is possible to observe a high contrast between sputtered vs nonsputtered areas, likely indicating carbon deposition during the reaction. The present study suggests that the deactivation mechanism of δ-Ni_5_Ga_3_ is a combination of crystal phase change and carbon deposition, where the last one could be promoted by the as-formed surface phases of Ni_13_Ga_9_ and Ni_2_Ga_3_, or by the pure Ni particles.

The catalyst ability to reactivate by exposing it to H_2_ at high temperatures is definitely a good quality for a possible future industrial application. Nevertheless, better than a catalyst that can reactivate is one that does not deactivate. The experiment shown in Figure 4c was performed to probe the long-term catalyst stability. When kept under reaction gases under milder conditions (165 °C), the thin film exhibits high stability, losing only about 5% of the initial activity over 35 h. A temperature of 165 °C was selected as it is close to the maximum turnover of the catalyst toward methanol. At 185 °C, the activity toward methanol decreases faster compared to 165 °C (Figure S19), and it is characterized by an increase over time of CH_4_ production, indicating that the catalyst top surface might be already slowly alloying to the unwanted crystal phases, causing more Ni to be on the surface and therefore, increasing the selectivity to CH_4_. This is not the case for the experiment of Figure 4c, where the methanol selectivity stays constant over the whole experiment, see Figure S20.

## 4 Conclusions

In summary, the investigation of δ-Ni_5_Ga_3_ thin films for CO_2_ hydrogenation has yielded significant information on their catalytic properties. The synthesis method employed in this study allowed the production of well-defined δ-Ni_5_Ga_3_ thin films at considerably lower temperatures and higher purity compared to any other methods used so far in the literature. It was possible to confirm that the catalyst is active independently of the presence of other phases, which could instead act as spectators of the reaction or just influence its selectivity.

By their nature, thin films have very little influence from their support. This study proves that the δ-Ni_5_Ga_3_ catalyst is active for methanol synthesis by itself, even when the support is not exposed to the reaction environment and metal–support interactions are minimized. The catalyst shows high activity and selectivity at lower temperatures compared to the literature, likely due to the higher purity of the δ phase (and the absence of, e.g., the  phase). This study sets an example of the potential of magnetron sputtering for the synthesis of complex catalysts compared with traditional chemical approaches. Using this information and employing this method in future studies could help to reach an even deeper knowledge of the system.

Furthermore, magnetron sputtering and thin films allowed a precise postreaction GI-XRD measurement that would have otherwise not been possible on other forms of the same catalyst. The clarification of the deactivation mechanism, particularly the reversible nature of the crystal phase change from δ-Ni_5_Ga_3_ to other ratios of the Ni/Ga system, represents a notable advancement in understanding the catalyst behavior. These findings not only contribute to the fundamental understanding of δ-Ni_5_Ga_3_ as a catalyst for methanol production but also pave the way for future research aimed at optimizing its performance and stability for industrial applications.

Moving forward, future studies could focus on understanding the reaction mechanism of this physically synthesized model system given these new insights. The versatility of thin films makes this system ideal for in situ/operando analysis with different techniques. High-pressure XPS and in situ XAS could give a clear indication of the state of the surface under the reaction conditions. The role of Ga_2_O_3_, for example, could be tackled more easily in such a system compared with others where the catalyst purity is not as high. Lastly, a comprehensive study on physically synthesized size-selected nanoparticles would also help to elucidate the catalyst active site necessary to ensure high activity and selectivity toward methanol.

## Supporting Information Available

Literature comparison of the synthesis of δ-Ni_5_Ga_3_; materials information; thin film preparation and handling after deposition; extra data and measurements of XPS, ISS, EDS, XRR, GI-XRD, and SEM; gas calibration in the QMS (PDF)
