# Supplementary material for: Magnetron Sputtering of Pure δ-Ni5Ga3 Thin Films for CO2 Hydrogenation
Source: ACS Catal. 2024 Aug 6;14(16):12592–601. doi: 10.1021/acscatal.4c03345 (PMC11334101; doi:10.1021/acscatal.4c03345)
Supplement: Supplementary file 1 — cs4c03345_si_001.pdf [file cs4c03345_si_001.pdf]

Supporting Information: Magnetron Sputtering of Pure δ-Ni_5Ga_3 Thin-Films for CO_2 Hydrogenation

Filippo Romeggio[1*], Jonathan F. Schouenborg[1], Peter C. K. Vesborg[1], Ole Hansen[2], Jakob Kibsgaard[1], Ib Chorkendorff[1], Christian D. Damsgaard[1,2*]

[1]DTU Physics, Technical University of Denmark, Kongens Lyngby, DK-2800, Denmark.
[2]DTU Nanolab, Technical University of Denmark, Kongens Lyngby, DK-2800, Denmark.

*Corresponding author(s). E-mail(s): filro@dtu.dk; cdda@dtu.dk;
Contributing authors: jofsch@dtu.dk; peter.vesborg@fysik.dtu.dk; ohan@dtu.dk; jkib@fysik.dtu.dk; ibchork@fysik.dtu.dk;

Contents

1 Literature synthesis comparison **3**

2 Materials **4**
2.1 δ-Ni_5Ga_3 4

3 Thin-films preparation **5**
3.1 μ-reactor production 5
3.2 Si wafer dummies 6
3.3 Surfaces pre-treatment 6
3.4 Handling after deposition 7

4 Characterization **9**
4.1 As-deposited films 9
4.1.1 XPS 9
4.1.2 ISS 11
4.1.3 EDS 12
4.1.4 XRR 13
4.2 Annealed films 14
4.2.1 Non-normalized GI-XRD 14
4.2.2 SEM 15
4.3 Activity measurements 16
4.3.1 Isotope corrections 19
4.4 Deactivation and stability 21
4.5 SiO_2 substrate 25

5 QMS and calibration **26**

S1 Literature synthesis comparison

Table S1 shows a comparison with all the papers published on Ni_5Ga_3 for CO_2 to methanol and their synthesis conditions to form the desired δ phase. The synthesis reported in our study allows to synthesize this phase with a ΔT of around 200°C compared to all other techniques (except for CCE, where ΔT = 115°C).

Method	Synthesis condition	Reference and year
IWI	700°C in H_2 for 2h	[1], 2014
IWI	700°C in H_2 for 2h	[2], 2014
CP	600°C in H_2 for 7h	[3], 2017
CCE	500°C calcination for 6h + $NaBH_4$ in ethanol	[4], 2017
IWI	630°C in H_2 for 2h	[5], 2018
IWI	500°C calcination for 6h + $NaBH_4$ in ethanol	[6], 2019
IWI	400°C calcination for 4h + 700°C in H_2 for 2h	[7], 2019
CCE	500°C calcination for 6h + $NaBH_4$ in ethanol	[7], 2019
Metal melting	melting at 1500°C	[8], 2019
IWI	500°C calcination for 3h + $NaBH_4$ in ethanol	[8], 2019
CCE	500°C calcination for 6h + $NaBH_4$ in ethanol	[8], 2019
IWI	400°C in N_2O + 550°C in H_2 for 3h	[9], 2019
IWI	700°C in H_2 for 2h	[10], 2020
IWI	700°C in H_2 for 6h	[11], 2020
Urea hydrolysis	700°C in H_2 for 6h	[11], 2020
IWI	680°C in H_2 for 1h	[12], 2020
CP	700°C in H_2 for 2h	[13], 2020
CP	400°C calcination for 2h + 600°C/800°C in H_2 for 2h	[14], 2021
CP[a]	700°C calcination for 2h + 600°C in H_2 for 1h	[15], 2021
E-beam evaporation	Not characterized	[16], 2021
IWI	400°C calcination for 4h + 700°C in H_2 for 2h	[17], 2021
CP	400°C calcination for 4h + 700°C in H_2 for 2h	[17], 2021
CCE	500°C calcination for 6h + $NaBH_4$ in ethanol	[17], 2021
IWI	500°C calcination for 2h + 500°C in H_2 for 1h	[18], 2021
IME	470°C calcination for 6h + 700°C in H_2 for 7.5h	[19], 2022
CP	600°C in H_2 for 7h	[20], 2023
Co-grafting	600°C in H_2 for 12h	[21], 2024
IWI	400°C calcination for 4h + 700°C in H_2 for 3h	[22], 2024
IWI	500°C calcination for 2h + 550°C in H_2 for 1h	[23], 2024
Ball milling	500°C calcination for 2h + 600/700°C in H_2 for 2h	[24], 2024
**Magnetron Sputtering**	**385°C in H_2 for 1h**	**This work, 2024**

Table S1: IWI = Incipient Wetness Impregnation, CP = Co-Precipitation, CCE = Co-Condensation-Evaporation, IME = Inverse Micelle Encapsulation. [a]Surfactant-assisted.

S2 Materials

Nickel/Gallium target (99.9+% purity, 62.5/37.5 at%) was purchased from Kurt J. Lesker Company. CO_2, H_2, and Ar gases used during catalytic activity testing were purchased as 6.0 purity gases. Ar and He used for depth profiling XPS and ISS were also 6.0 purity.

S2.1 δ-Ni_5Ga_3

Below is an image showing all the thermodinamically stable phases of the Ni/Ga system. The data are downloaded from materials project [25].

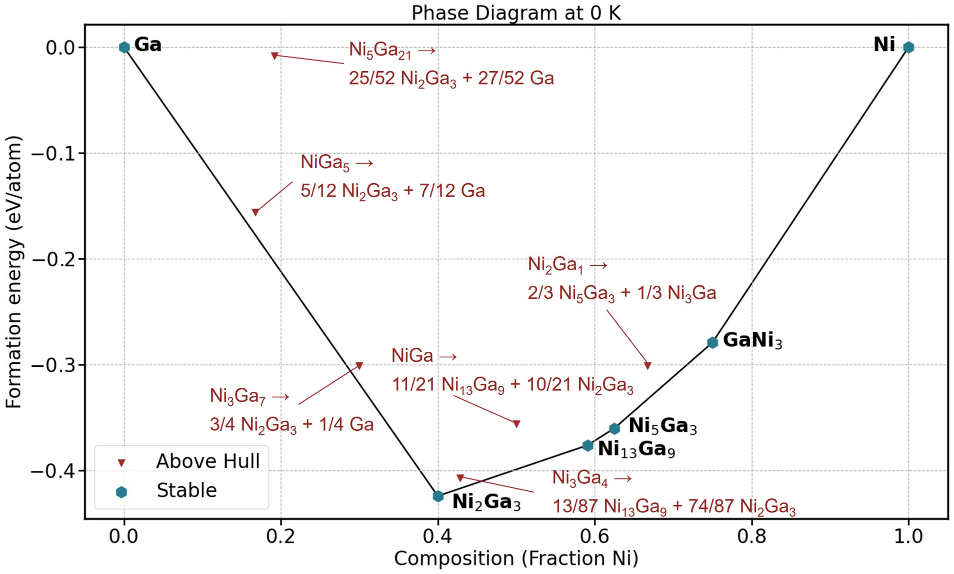

Fig. S1: **Thermodynamic stability of the Ni/Ga system** Calculated thermodynamic stability of different crystal phases of the Ni/Ga system. In red are represented phases that are expected to decompose to stable phases as shown in the plot.

S3 Thin-films preparation

S3.1 μ-reactor production

The μ-reactors are state-of-the-art devices fabricated in the DTU Nanolab cleanroom. They are produced following a series of steps involving oxide growth on a Si (100) wafer, lithography, etching, and dicing. The reactor bed consists of 50 nm SiO_2 which is grown thermally to ensure the reactor is inert to most reactions at the usual operating temperatures (20-450°C). The μ-reactor geometry and schematics is available in Figure S2. A SEM image of the μ-reactors surface is also shown below (Figure S3).

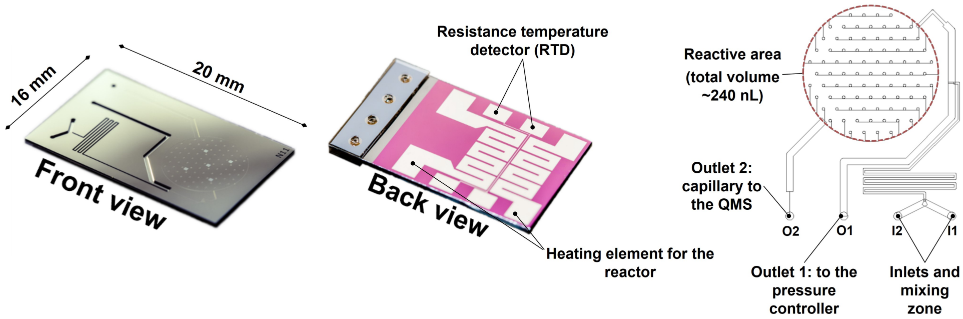

Fig. S2: **μ-reactor front/back and its schematics** The central reactive area is 3 μm deep. The total reactor volume (red dotted area) is around 240 nL. Image reproduced from [26].

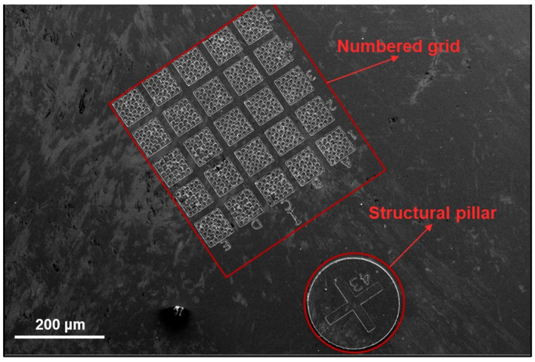

Fig. S3: **SEM image of a pristine μ-reactor surface** The round, numbered feature is one of many pillars that are present on the reactor surface to prevent the top Pyrex lid to collapse on the 3 μm thin reaction area. The grid is used for identical location imaging pre-/post-catalytic activity experiments.

S3.2 Si wafer dummies

Si wafers were used as dummy samples for parallel characterization of the deposited thin-films. The Si wafers were treated in the cleanroom exactly the same way as the μ-reactors to ensure consistency between the two substrates and making the characterization solid and reliable. The dummy chips, therefore, fully corresponds to the μ-reactors, having the same layer thicknesses and differing only for their geometry. A photo of the dummy chips and their schematics is given in Figure S4.

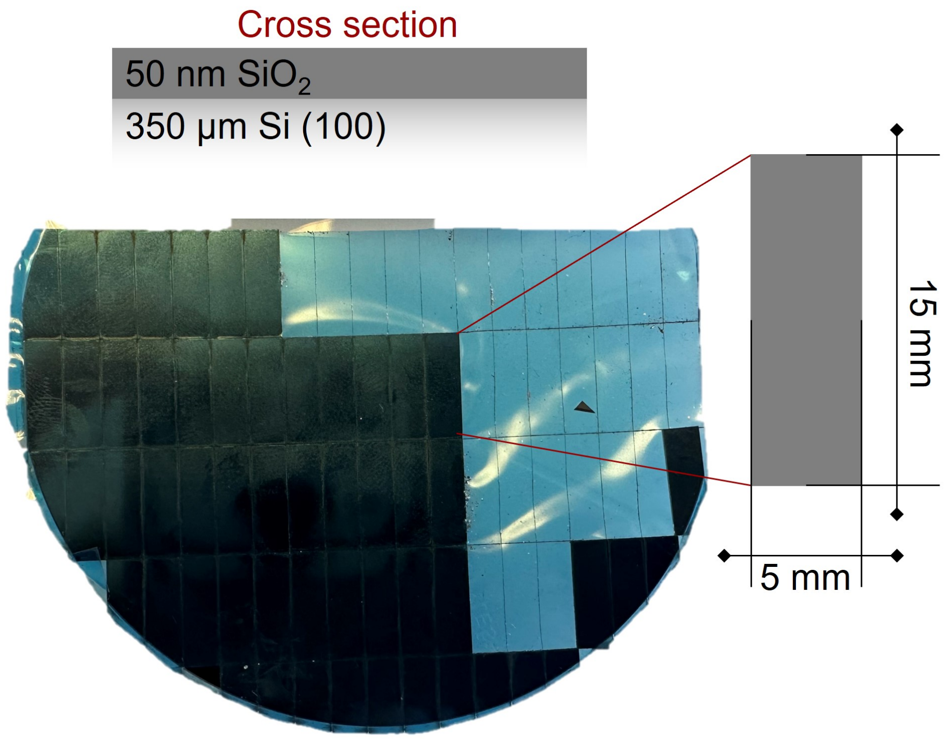

Fig. S4: **Overview of the dummy wafers as received from the cleanroom** The blue tape on top of the wafers was kept on from the cleanroom fabrication in order to ensure maximum cleanliness. To remove any contamination from the tape itself, the wafers were wiped with ethanol before deposition.

S3.3 Surfaces pre-treatment

Both the μ-reactors and the dummy chips were treated before being introduced in the UHV magnetron sputtering for deposition. The pre-treatment, which consists in a simple ethanol wiping and Ar^+ plasma cleaning, is necessary to remove residues from the polymer on the blue tape that seals the diced μ-reactors/Si wafers from the

cleanroom fabrication. If not cleaned, the surfaces would look as reported in the figure below, potentially influencing material deposition and catalytic activity.

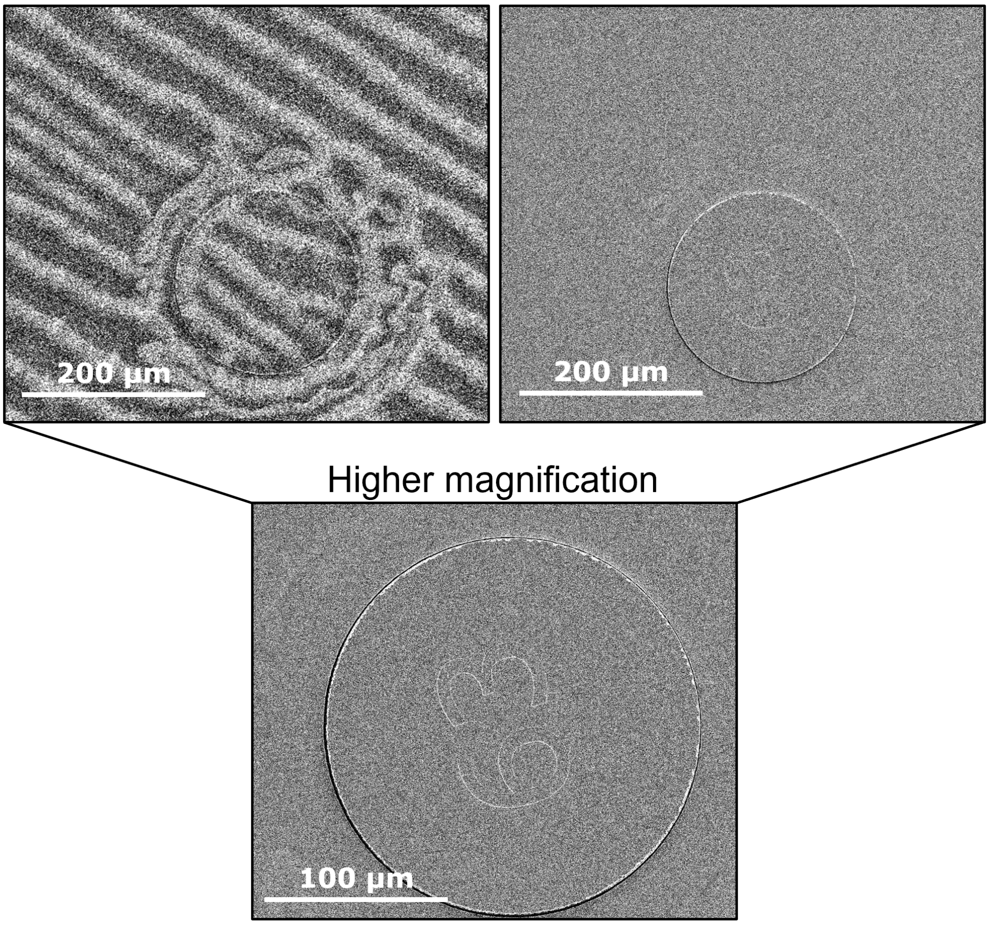

Fig. S5: **Effect of reactor cleaning** SEM images of the reactor surface when not pre-cleaned (left) and when cleaned (right). The images where taken at identical location (pillar 63).

S3.4 Handling after deposition

Immediately after deposition, the samples are removed from the magnetron sputtering chamber and follow three possible paths in order to minimize air exposure:

1. Anodic bonding. This step is performed to seal the reactor open surface from atmosphere, creating a close environment, and allowing gases to flow. It is run at high temperatures and 1kV. A more detailed explanation is reported in [26–28].

2. Characterization. Usually, one magnetron sputtering session allows for the parallel deposition on multiple samples. Therefore, some of the as-deposited films on the μ-reactors (or dummy chips) are immediately characterized in parallel with different techniques. This is performed to obtain solid results and avoiding having time as a possible parameter to consider in the data analysis. For catalytic testing, the optimum gas composition was initially tested and fixed to H_2:CO_2=3:1 (Figure S6).

3. Long-term storage. In case the samples are not immediately used for either catalytic testing or characterization, they usually are stored in a vacuum chamber attached to a dry roughing pump. The samples are inserted in the vented chamber, which is then pumped down and flushed with Ar $\geq$3 times to ensure low contamination levels from atmosphere.

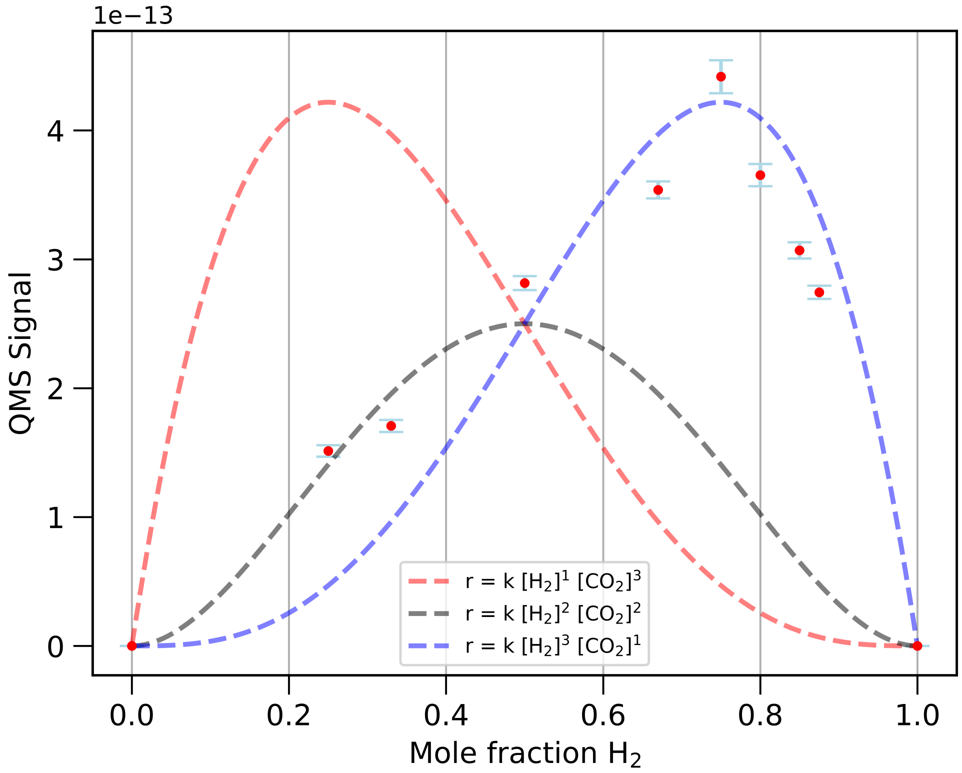

Fig. S6: **Inlet gas composition dependence** Catalytic activity towards methanol depending on the inlet ratio of H_2/CO_2. The testing conditions are 1 bar and 175°C. The data are averaged from different experiments, which showed consistently an optimum ratio of 3:1.

S4 Characterization

S4.1 As-deposited films

4.1.1 XPS

For the quantification of Ni2p and Ga2p shown in Figure 2 in the manuscript, the following parameters were used:

- Shirley background subtraction
- Ni2p sensitivity factor: 20.765
- Ga2p sensitivity factor: 33.500
- Peak library: ALTHERMO1

The Peak Area was corrected for the total dwell time per channel, number of scans and energy channel width:

$$NormalisedPeakArea = \frac{PeakArea}{SF \cdot TXFN \cdot ECF}$$

Where:

- $TXFN$ = transmission function
- SF = sensitivity factor
- ECF = energy compensation factor

The ECF value was set as an averaged value derived from the Tanuma, Powell, Penn 2 Method (TPP-2M) [29].

The atomic concentration of each peak was calculated as: Normalised Area * 100 / Sum of Normalised Areas. The areas were taken from the same etching level for Ni and Ga (etching level 9, 90 seconds etching).

The transmission function was calculated from a polynomial fit of:

$$log\left[\frac{PeakArea}{PE \cdot XSF}\right] \text{ vs. } log\left[\frac{KE}{PE}\right]$$

Where:

- PE = Pass Energy;
- KE = Kinetic Energy
- XSF = Relative sensitivity factor applied to normalise the two curves (mainly due to photoelectron cross section differences of distinct orbitals).

The survey spectrum for the first and last etching level is given in Figure S7. Charge shift correction was performed using C1s = 284.8eV as calibration peak.

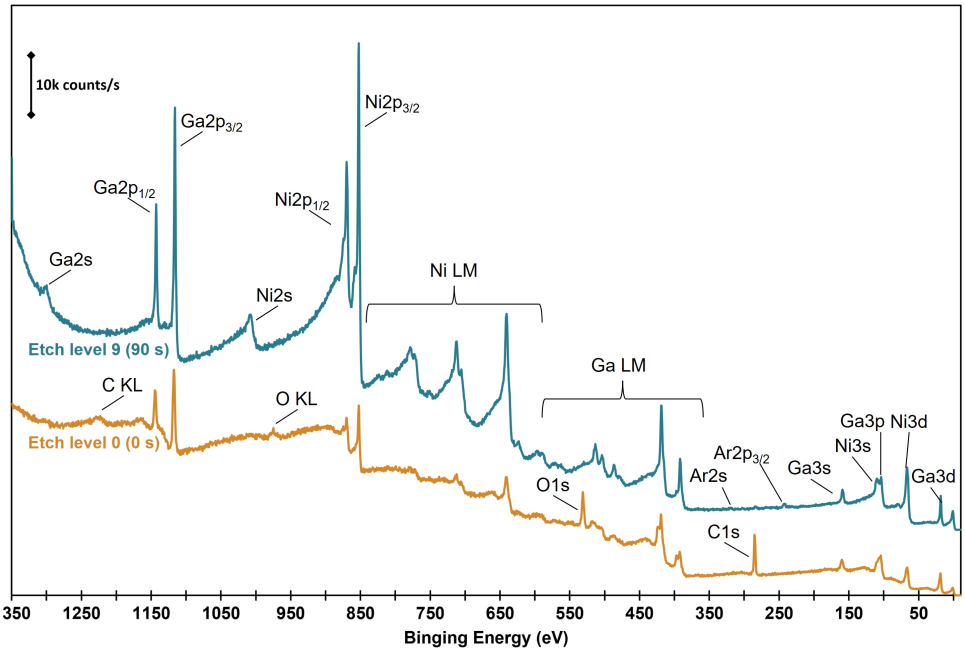

Fig. S7: **XPS survey spectra** Survey spectra of one of the δ-Ni_5Ga_3 samples at etching cycle 0 and 9.

4.1.2 ISS

Figure S8 shows ISS spectra of the as-deposited catalyst. After some etching cycles, only 3 peaks are present: Ni, Ga, and some residual oxygen. This serves as further indication on the purity of the surfaces deposited through magnetron sputtering.

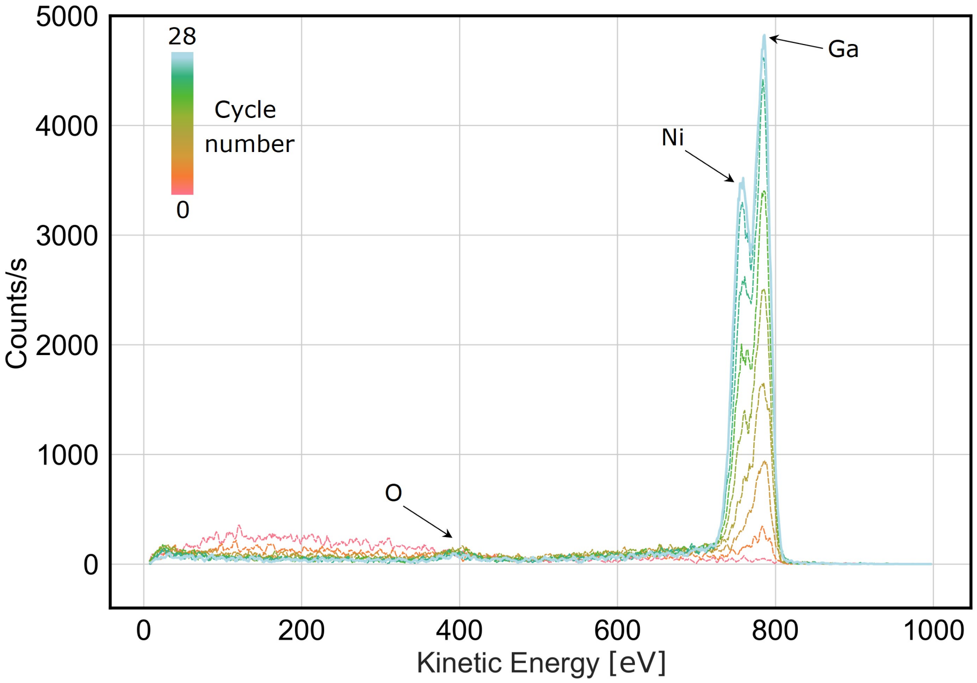

Fig. S8: **Ion Scattering Spectroscopy of the as-deposited δ-Ni_5Ga_3** Ion Scattering Spectroscopy of the as-deposited δ-Ni_5Ga_3. The presence of only two peaks indicate the high purity of the sample surface. The measurement was done at 1kV.

4.1.3 EDS

AZtec software was used for quantification of Ni, Ga, O, C, and Si. The measurement was taken with the following parameters:

- Scanning type: Point&ID
- dead time: 30%
- 2048 channels
- energy per channel: 10eV
- process time: 5
- pixel dwell time: 120μs
- strobe resolution: 34 eV

Ni was quantified with K series lines and a fit index of 1.3, while Ga with L series lines and a fit index of 0.7. Energy calibration was performed with Cu K series.

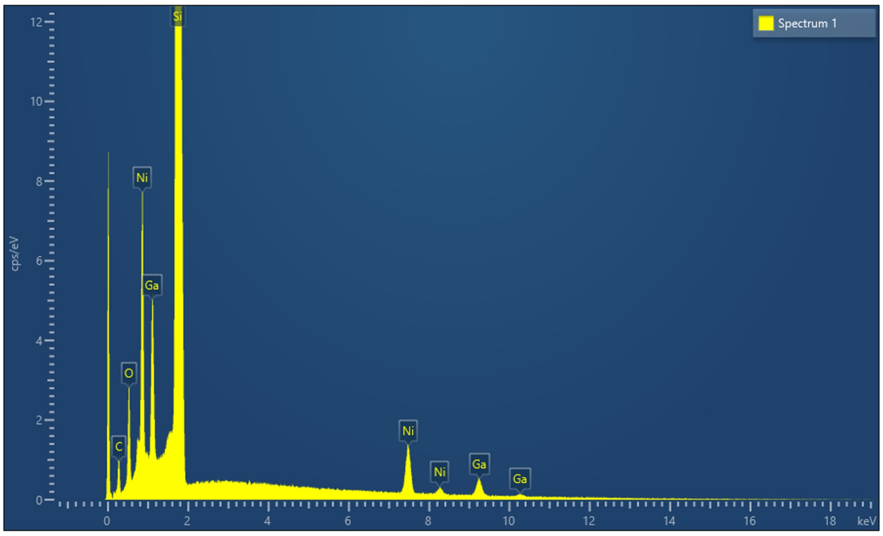

Spectrum Label	Atomic %
**C**	19.20
**O**	7.94
**Si**	69.34
**Ni**	2.16
**Ga**	1.37
**Total**	**100.00**

Theoretical Ni/Ga ratio in Ni_5Ga_3: Ni/(Ni+Ga) ~ **0.625**

Sample Ni/Ga ratio from EDS: Ni/(Ni+Ga) ~ **0.612**

Fig. S9: **EDS spectrum of a δ-Ni_5Ga_3 sample** EDS spectrum and quantification of a δ-Ni_5Ga_3 sample, showing a comparable bulk ratio of Ni/Ga to the expected 5/3.

4.1.4 XRR

The XRR analysis was performed using the expected values of the thickness of the thin-films as initial parameter (50 nm). The initial density value was set to the one of the bulk Ni_5Ga_3 crystal phase (9.08 g/cm^3, see mp-11398 structure on Materials Project [25]) while roughness was set to 1 nm as starting value. The fitting was first performed using the genetic fit from the aforementioned starting parameters and afterwards fitted with the segmented fit. For testing the stability of the fits, the starting parameters were changed to around 10% lower values to confirm that the same conversion occurred. In Figure S10 are the raw data and fits from two more samples of as-deposited δ-Ni_5Ga_3 thin-films.

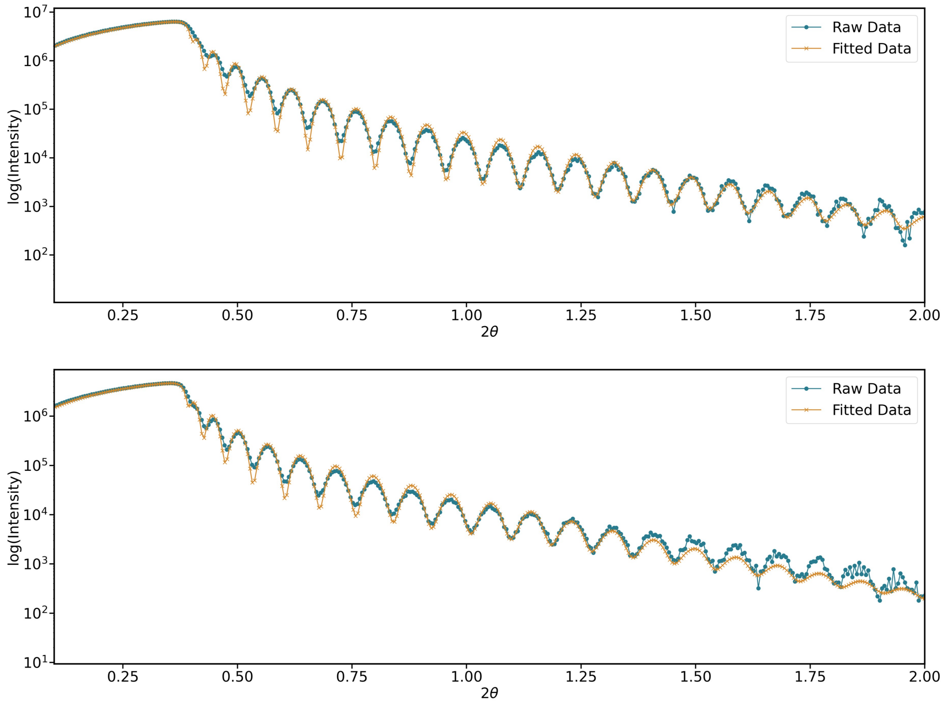

Fig. S10: **XRR fitting of as-deposited δ-Ni_5Ga_3 thin-films** For determination of the uncertainty of the XRR results, 3 thin-films were tested and analysed.

S4.2 Annealed films

4.2.1 Non-normalized GI-XRD

For a more clear comparison of the crystal and phase growth after annealing with hydrogen at 385°C, below is reported a figure showing the non-normalized XRD data.

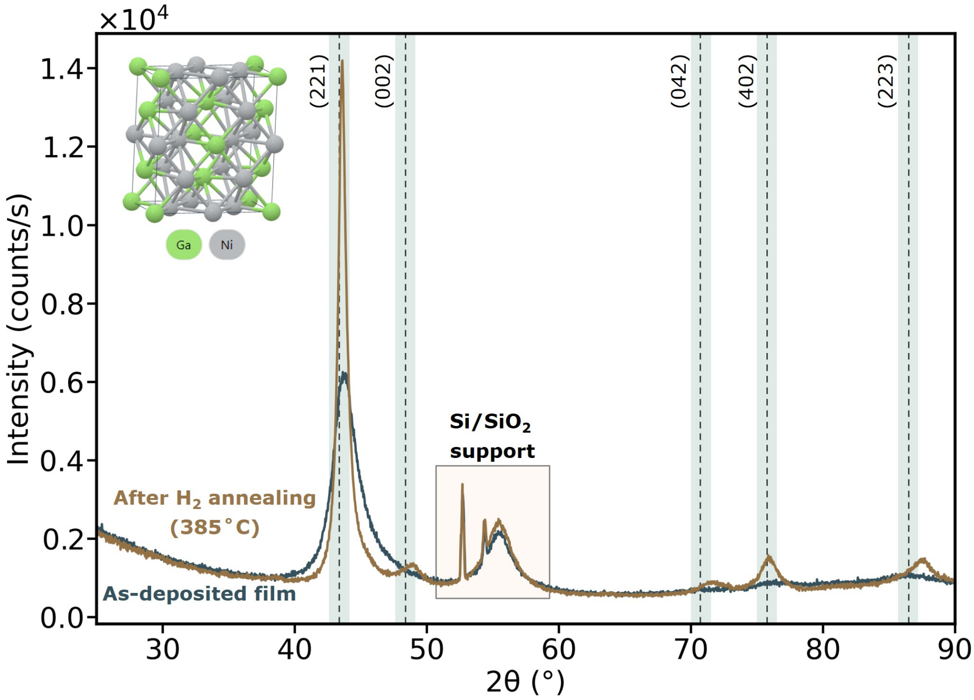

Fig. S11: **XRD of δ-Ni_5Ga_3 before and after H_2 annealing** Non-normalized XRD plot of a δ-Ni_5Ga_3 sample before and after hydrogen annealing. This representation helps understanding the crystal growth after annealing.

4.2.2 SEM

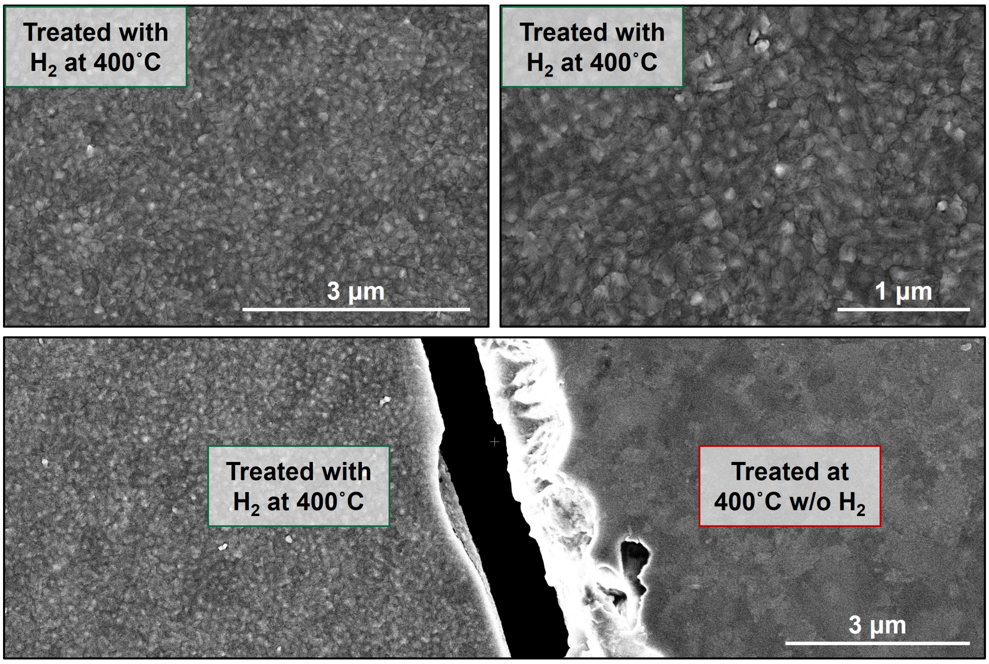

Fig. S12: **SEM images of the samples surfaces after activation** SEM images showing the activated sample surface. The bottom image shows a part of the sample that was exposed to high temperature but not to hydrogen, showing that not only temperature, but also hydrogen, is necessary for crystal growth.

S4.3 Activity measurements

Below are some supporting figures and information regarding the catalytic activity of the δ-Ni_5Ga_3 catalyst. The experiments were performed at an activation temperature of 385°C or 400°C interchangeably, since it did not affect the catalytic activity.

Table S2 summarizes the main reaction indicators in the region of interest for methanol synthesis. Since the CO signal in the QMS is around 3 orders of magnitudes above that one of Methanol and Methane (mass 28 is a cracking pattern of CO2, which is fed as reagent), the selectivity was calculated as "CO free" as done in previous studies [1, 2]. The results are quite close to those shown by Studt *et at.* [1], where the reaction was also conducted at 1 bar. In that study, a (CO free) selectivity of methanol + dimethyl ether approaching 100% was observed. Excluding dimethyl ether, the results would be even closer to those obtained with the μ-reactors.

**Temperature (°C)**	**Selectivity (%)**	**Conversion (%)**	**Space-Time Yield (μmol$_{MeOH}$/L/h)**	**Activity (μmol$_{MeOH}$/m$^2{}_{cat}$/h)**
135	77	Below detection	$2.3 \cdot 10^2$	1.1
160	82	0.5	$4.6 \cdot 10^2$	2.2
185	76	1.1	$4.8 \cdot 10^2$	2.4
210	61	2.5	$2.5 \cdot 10^2$	1.2

Table S2: Summary of the reaction indicators at the relevant temperatures for methanol synthesis. The selectivity was calculated without accounting for CO.

Table S3 presents a comparison of catalytic activity at the top performance temperature for Ni_5Ga_3 across various literature sources. This comparison includes all the published studies that tested the catalyst under identical conditions as ours (1 bar, H_2:CO_2=3:1). The GHSV of the μ-reactors is considerably lower compared to the ones of other setups, meaning that very little gas flows per unit of time (ensuring high product sensitivity). Comparisons of production rates are only valid when conducted under conditions far from equilibrium. In this study, this happens only at very low temperatures, at which other studies often report no observable catalytic activity (T $\leq$ 160°C). Moreover, given that this catalyst is still relatively new and yet not well understood, variations in the normalization of active sites and surface areas contribute to significant uncertainty in the reported activity. Consequently, the observed methanol production rates are very different across different studies and cross-comparisons remain very difficult. In this specific case, the only published study which shows results consistent with ours is the one reported by Cuenya *et al.*, with an activity of 0.11 μmol$_{MeOH}$/g$_{cat}$/h.

**Highest activity T (°C)**[a]	**Activity ($x_{MeOH}/y_{cat}/t$)**	**GHSV (h^{-1})**	**Reference**
210	0.25 mol/mol/h	$1.47 \cdot 10^4$	[1]
210	0.24 g/g/h	$1.55 \cdot 10^4$	[2]
200	38.85 μmol/g/min	$7.33 \cdot 10^3$	[7]
250	32.3 μmol/m^2/h	$7.33 \cdot 10^3$	[10]
220	0.11 μmol/g/h	$8.15 \cdot 10^3$	[19]
**185**	**0.1-5 μmol/g/h**[b]	**$0.18 \cdot 10^3$**	**This work**

Table S3: Literature overview of the Ni_5Ga_3 catalysts tested at 1 bar. [a]The reported temperature corresponds to the condition of maximum activity for that specific study. [b]Considering the entire mass of the deposited film (50 nm) vs only the top active volume (1 nm). GHSV = Gas Hourly Space Velocity.

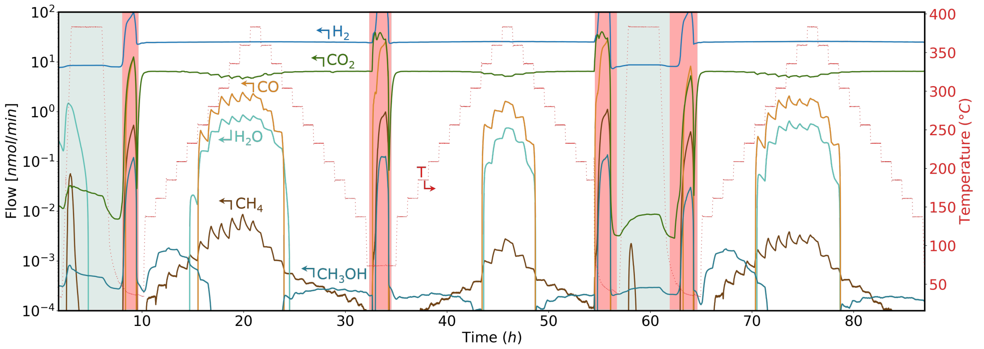

Fig. S13: **Full activity plot with treated data** Treated catalytic activity plot corresponding to Figure 3a in the manuscript. The data were corrected for background, temperature, cracking masses, and isotopes contributions.

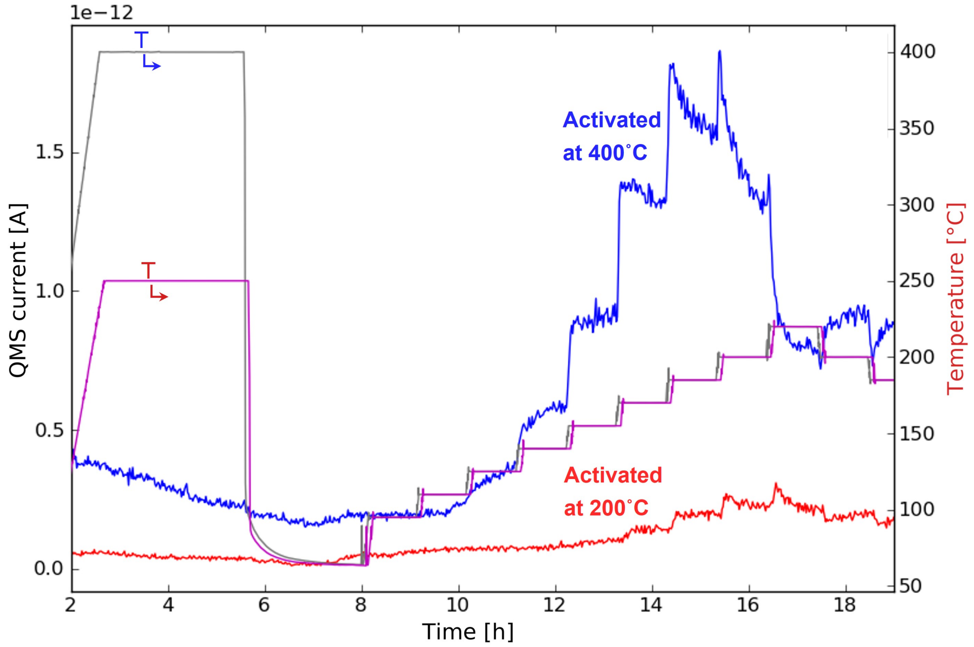

Fig. S14: **Activation temperature influence** QMS raw data showing the influence of temperature activation in H_2 on the catalytic activity towards methanol.

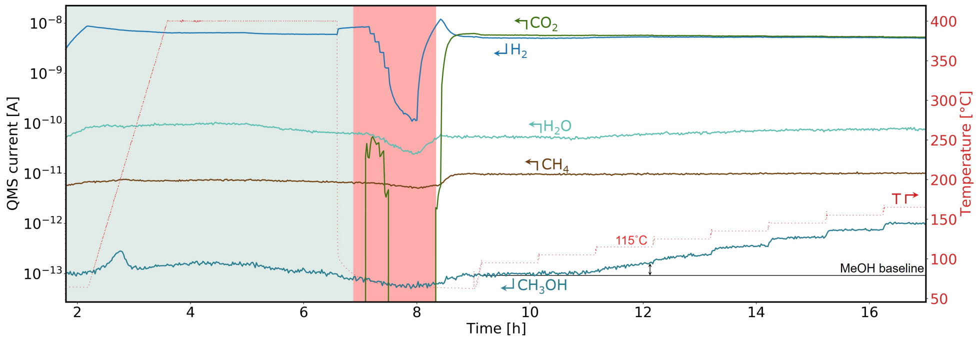

Fig. S15: **Catalytic activity of δ-Ni_5Ga_3 at low temperatures** QMS raw data of a δ-Ni_5Ga_3 thin-film sample showing methanol production at temperatures as low as 115°C.

4.3.1 Isotope corrections

Since the μ-reactors are extremely sensitive to products, it is relevant to discuss the corrections implemented when analyzing (and plotting) catalytic activity figures. As visible in Figure 2A in the main paper, it seems that at high temperatures some methanol is produced. This interpretation would be wrong, because the plot was not corrected for possible isotopes. Carbon and oxygen present several isotopes. Here are reported the most abundant:

- C^{12} = 98.84 %
- C^{13} = 1.06 %
- O^{16} = 99.74 %
- O^{18} = 0.21 %

From the abundance of different isotopes, it is clear that also mass 29 and 30 would be expected together with mass 31. Figure S16 shows that at high temperatures, mass 29, 20, and 31 seem to grow together with mass 28 (CO) and proportionally to their probability, confirming the hypothesis and ensuring that M31 at those temperatures must not be considered as methanol.

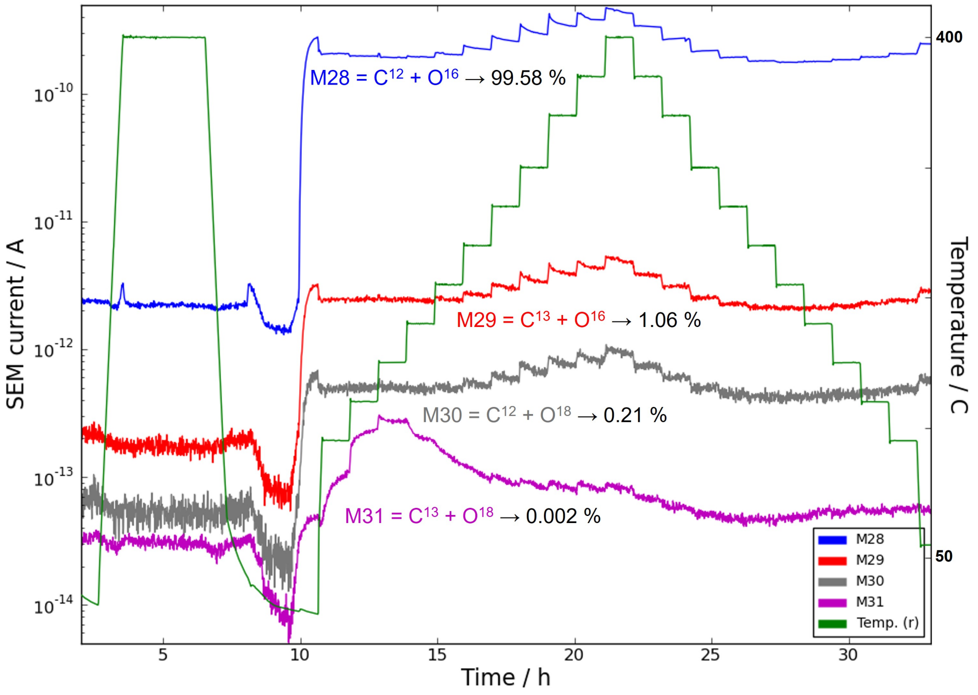

Fig. S16: **Mass 28, 29, 30, and 31 scan** Raw catalytic activity plot showing the scanned masses 28, 29, 30, and 31. The presence of all those masses confirms that the M31 signal at high temperatures correspond to an isotope of CO and not to methanol. This plot is particularly helpful to understand the selectivity of the catalyst. Since M18 and M28 have a background baseline which is approximately 3 orders of magnitude higher than the one of M31 (CH_3OH) and M15 (CH_4), H_2O and CO signals in this figure should be regarded as highly imprecise/with very high error bars.

S4.4 Deactivation and stability

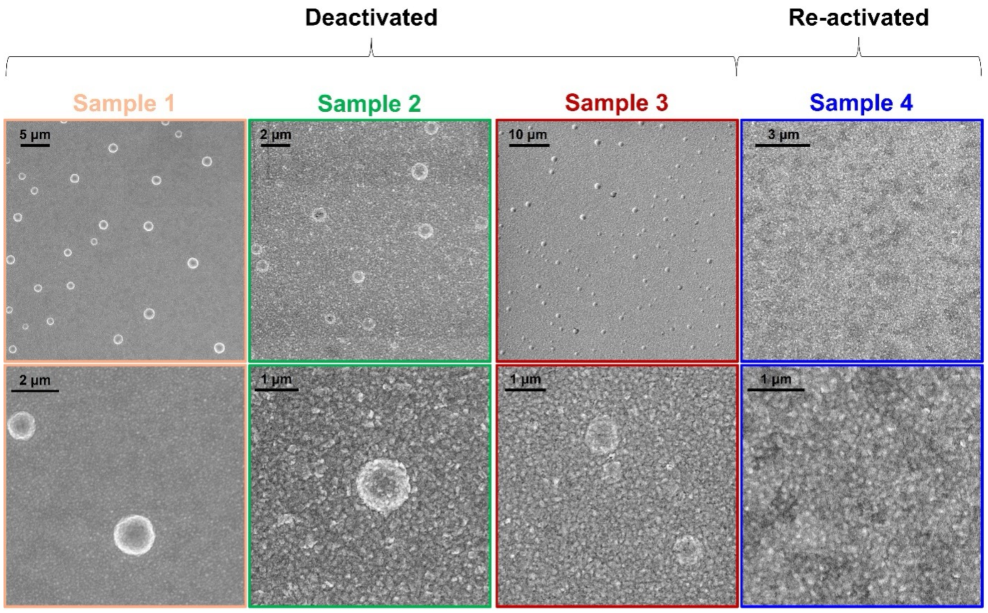

Fig. S17: **SEM images of the samples surface after deactivation** SEM images showing that all samples display the same behavior: round features are formed on the surface when the catalyst is deactivated. After reactivation (>385°C in H_2), the round features are not present on the sample anymore on most part of the reactive surface.

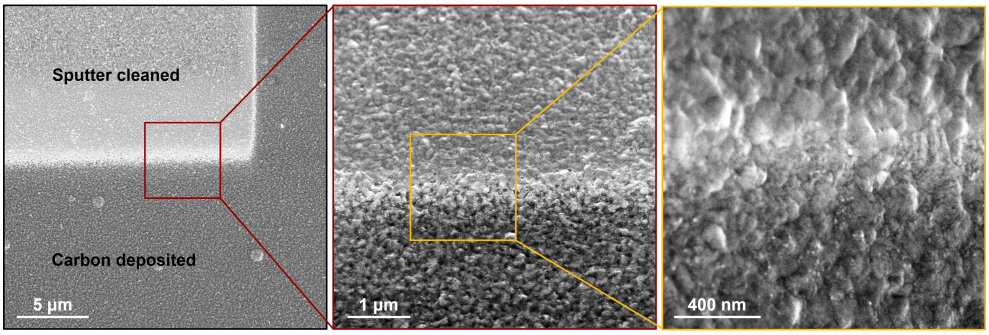

Fig. S18: **FIB-SEM on a deactivated sample showing carbon deposition.** SEM images of a deactivated sample to show round features + carbon deposition. The surface was quickly sputtered with an Ar Focused Ion Beam (FIB) to remove the layer of carbon, enhancing the contrast between the two areas. Before imaging, the sample was exposed to reaction conditions and high temperatures for approximately 50 h to fully deactivate it and potentially promote carbon deposition to a higher extent. It is important to notice that the nature of the contrast could also be influenced from surface contamination and not entirely by the reaction deactivation process, since the sample was exposed to air after reaction and before mounting the sample in the SEM chamber.

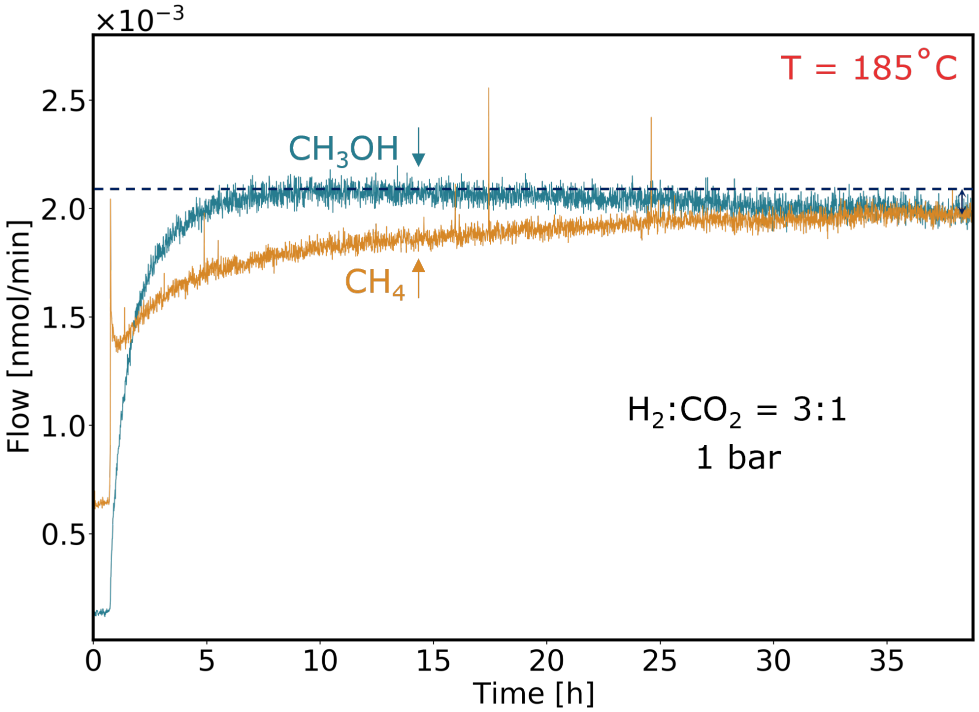

Fig. S19: **Long Time-On-Stream experiment to probe deactivation** Deactivation test at 185°C of a 50 nm δ-Ni_5Ga_3 thin-film, showing that the selectivity slowly shifts towards methane production as the catalyst deactivates.

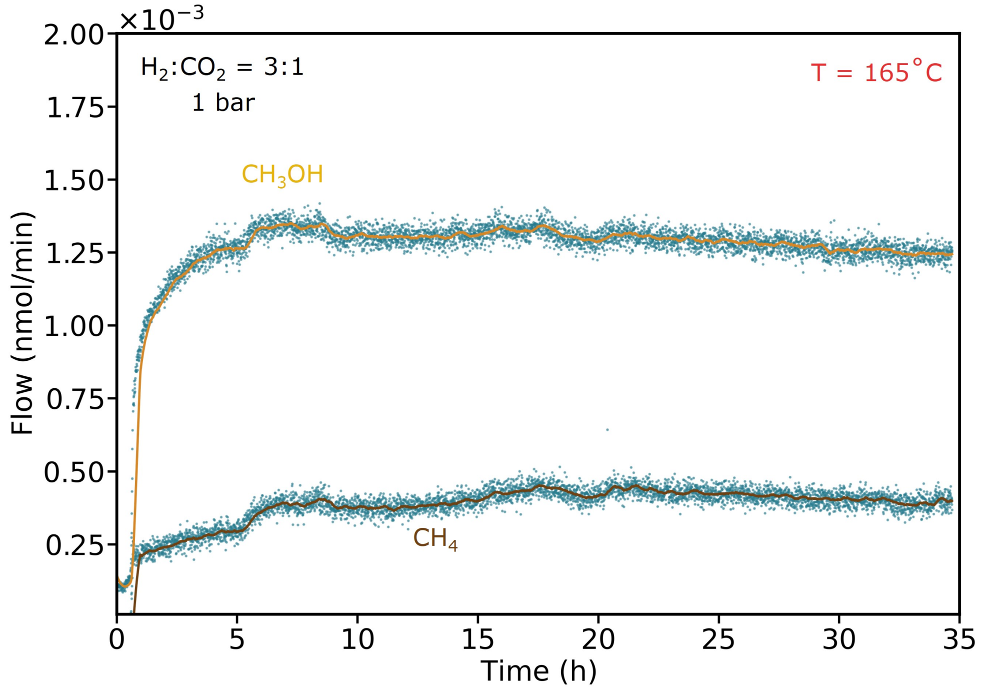

Fig. S20: **Long Time-On-Stream experiment to probe deactivation** Deactivation test at 165°C of a 50 nm δ-Ni_5Ga_3 thin-film. At lower temperatures (below 185°C) the catalyst is not undergoing any deactivation, and methanol selectivity does not change.

S4.5 SiO_2 substrate

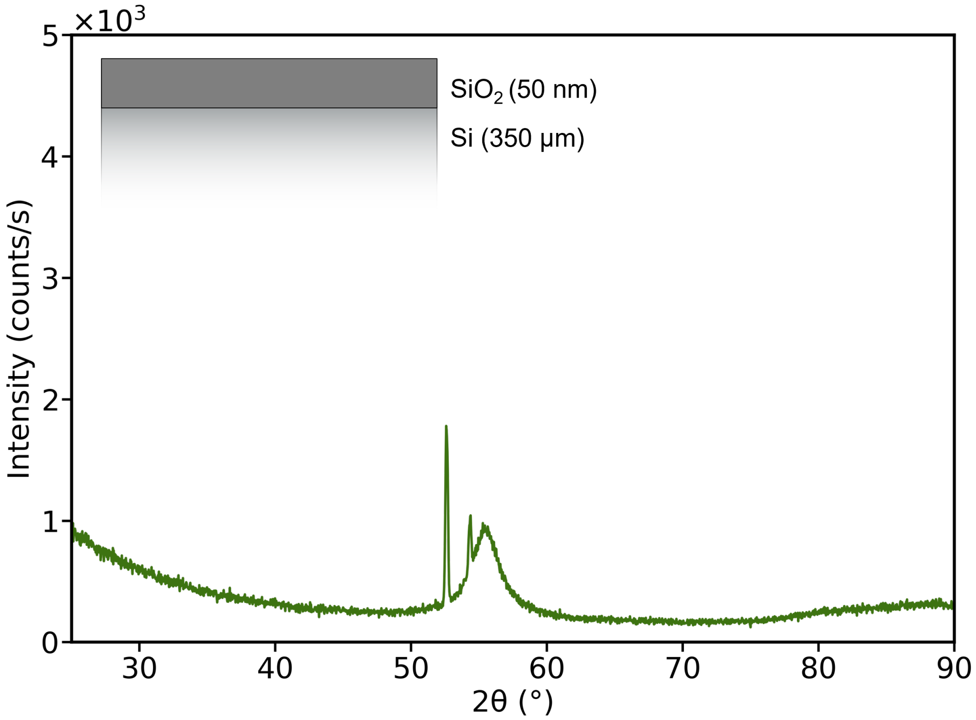

Fig. S21: **XRD of the pristine Si/SiO_2 support** The measurement was taken on the Si wafer support before any catalyst deposition, in order to disentangle some of the peaks in the diffractograms.

S5 QMS and calibration

The QMS measurements were conducted with a ionization energy of 70 eV, a SEM voltage of 1850 V, and an emission current of 0.1 mA. The different gases were detected in the QMS by scanning for the following masses: H_2 = M2, CO_2 = M44, H_2O = M18, CH_4 = M15, CH_3OH = M31. M15 and M31 were used for CH_4 and CH_3OH because they are the highest intensity cracking patterns. The raw signal was converted in a flow (nmol/s) after calibration with a baratron. In simple term, the baratron is a known volume connected to the outlet of the μ-reactor on the same line of the QMS. After the reactor is filled with 1 bar of pure gases, the valve to the QMS is closed, causing an increase of pressure in the line (and in the baratron). The pressure evolution is recorded and translated to a gas flow using the ideal gas law. For a more detailed explanation on the gas calibration, check reference [26]. After the calibration of CO_2, O_2, Ar, H_2, and He, the measured sensitivity factor can be plotted vs the calculated sensitivity factor through the following formula:

$$f_M^i = k \times \sigma_M^i \times \frac{I_M^i}{\sum_{M'} I_{M'}^i} \times T(M)$$

Where:

- k = normalization constant
- σ_M^i = ionization cross section
- I_M^i relative intensity of mass M
- $\sum_{M'} I_{M'}^i$ = intensity sum over all the cracking patterns originating from the analyzed molecule/gas
- $T(M)$ = transmission factor (usually $M^{-1/2}$)

After calculating the sensitivity factor for every molecule and plotting it against the measured one, it is possible to linearly interpolate the data (Figure S22). With it, it is possible to extrapolate the measured (actual) sensitivity factor for gases that cannot be flown in the reactor pure.

For transparency, the code utilized for one of the reactors can be seen and downloaded from the following link: https://github.com/filro/Ni5Ga3_Calibration.git

More information regarding the calibration method can be found in reference [30].

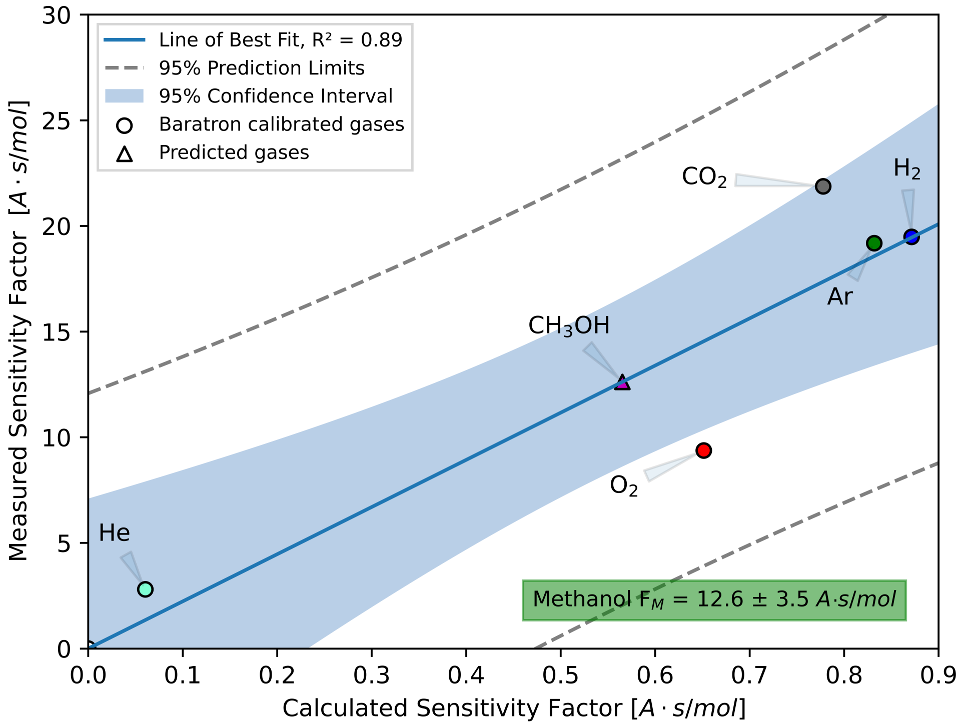

Fig. S22: **Calibration curve** Interpolated curve of calculated sensitivity factor vs measured/actual sensitivity factor (from the baratron measurements). With this plot, a methanol sensitivity factor of 12.6 ± 3.5 A·s/mol could be extrapolated.

References

[1] Studt, F., Sharafutdinov, I., Abild-Pedersen, F., Elkjær, C.F., Hummelshøj, J.S., Dahl, S., Chorkendorff, I., Nørskov, J.K.: Discovery of a Ni-Ga catalyst for carbon dioxide reduction to methanol. Nature Chemistry 2014 6:4 **6**(4), 320–324 (2014) https://doi.org/10.1038/nchem.1873

[2] Sharafutdinov, I., Elkjær, C.F., De Carvalho, H.W.P., Gardini, D., Chiarello, G.L., Damsgaard, C.D., Wagner, J.B., Grunwaldt, J.D., Dahl, S., Chorkendorff, I.: Intermetallic compounds of Ni and Ga as catalysts for the synthesis of methanol. Journal of Catalysis **320**(1), 77–88 (2014) https://doi.org/10.1016/J.JCAT.2014.09.025

[3] Chiang, C.L., Lin, K.S., Lin, Y.G.: Preparation and Characterization of Ni5Ga3 for Methanol Formation via CO2 Hydrogenation. Topics in Catalysis **60**(9-11), 685–696 (2017) https://doi.org/10.1007/s11244-017-0771-7

[4] Khanh Dieu Nguyen, H., Hong Dang, T., Le To Nguyen, N., Thi Nguyen, H., Thi Dinh, N., Chi Minh, H.: Novel Ni-Ga Alloy Based Catalyst for Converting CO 2 to Methanol (2017) https://doi.org/10.1002/cjce.23006

[5] Chen, P., Zhao, G., Liu, Y., Lu, Y.: Monolithic Ni5Ga3/SiO2/Al2O3/Al-fiber catalyst for CO2 hydrogenation to methanol at ambient pressure. Applied Catalysis A: General **562**, 234–240 (2018) https://doi.org/10.1016/J.APCATA.2018.06.021

[6] Nguyen, H.K.D., Dang, T.H.: Conversion of CO2 to methanol using NiGa/mesosilica (NiGa/MSO) catalyst. Journal of Porous Materials **26**(5), 1297–1304 (2019) https://doi.org/10.1007/s10934-019-00730-0

[7] Ahmad, K., Upadhyayula, S.: Conversion of the greenhouse gas CO2 to methanol over supported intermetallic Ga–Ni catalysts at atmospheric pressure: thermodynamic modeling and experimental study. Sustainable Energy & Fuels **3**(9), 2509–2520 (2019) https://doi.org/10.1039/C9SE00165D

[8] Khanh, H., Nguyen, D., Dang, H., Dinh, N.T., Huu, H., Nguyen, D., Toan, ., Duy, H.H., Affiliations, N.: Study on characterization and application of novel Ni-Ga based catalysts in conversion of carbon dioxide to methanol . Cite as: AIP Advances **9**, 85006 (2019) https://doi.org/10.1063/1.5116271

[9] Goyal, R., Lee, J., Sameer, S., Sarkar, B., Chiang, K., Bordoloi, A.: CNx stabilized Ni-Ga nanoparticles for CO 2 hydrogenation: Role of preparation methods (2019) https://doi.org/10.1016/j.cattod.2019.03.031

[10] Gallo, A., Snider, J.L., Sokaras, D., Nordlund, D., Kroll, T., Ogasawara, H., Kovarik, L., Duyar, M.S., Jaramillo, T.F.: Ni5Ga3 catalysts for CO2 reduction to methanol: Exploring the role of Ga surface oxidation/reduction on catalytic

activity. Applied Catalysis B: Environmental **267**, 118369 (2020) https://doi.org/10.1016/J.APCATB.2019.118369

[11] Men, Y., Fang, X., Gu, Q., Singh, R., Wu, F., Danaci, D., Zhao, Q., Xiao, P., Webley, P.A.: Synthesis of Ni5Ga3 catalyst by Hydrotalcite-like compound (HTlc) precursors for CO2 hydrogenation to methanol. Applied Catalysis B: Environmental **275**, 119067 (2020) https://doi.org/10.1016/J.APCATB.2020.119067

[12] Duyar, M.S., Gallo, A., Snider, J.L., Jaramillo, T.F.: Low-pressure methanol synthesis from CO2 over metal-promoted Ni-Ga intermetallic catalysts. Journal of CO2 Utilization **39**, 101151 (2020) https://doi.org/10.1016/J.JCOU.2020.03.001

[13] Ahmad, K., Upadhyayula, S.: Selective conversion of CO 2 to methanol over intermetallic Ga-Ni catalyst: Microkinetic modeling (2020) https://doi.org/10.1016/j.fuel.2020.118296

[14] Cortés-Reyes, M., Azaoum, I., Molina-Ramírez, S., Herrera, C., Larrubia, M.A., Alemany, L.J.: NiGa Unsupported Catalyst for CO2 Hydrogenation at Atmospheric Pressure. Tentative Reaction Pathways. Industrial and Engineering Chemistry Research **60**(51), 18891–18899 (2021) https://doi.org/10.1021/acs.iecr.1c03115

[15] Rasteiro, L.F., Rossi, M.A.L.S., Assaf, J.M., Assaf, E.M.: Low-pressure hydrogenation of CO2 to methanol over Ni-Ga alloys synthesized by a surfactant-assisted co-precipitation method and a proposed mechanism by DRIFTS analysis. Catalysis Today **381**, 261–271 (2021) https://doi.org/10.1016/J.CATTOD.2020.05.067

[16] Lin, P.-S., Chang, S.-T., Chen, S.-Y., Luh, D.-A., Wang, C.-H., Yang, Y.-W.: Hydrogenation of CO 2 on NiGa thin films studied by ambient pressure x-ray photoelectron spectroscopy. Journal of Physics D: Applied Physics J. Phys. D: Appl. Phys **54**(12pp), 424004 (2021) https://doi.org/10.1088/1361-6463/ac1370

[17] Ahmad, K., Anushree, Upadhyayula, S.: Deactivation behaviour of intermetallic Ga-Ni catalyst in CO2 hydrogenation to methanol. Greenhouse Gases: Science and Technology **11**(5), 1056–1065 (2021) https://doi.org/10.1002/GHG.2117

[18] Rasteiro, L.F., De Sousa, R.A., Vieira, L.H., Ocampo-Restrepo, V.K., Verga, L.G., Assaf, J.M., Da Silva, J.L.F., Assaf, E.M.: Insights into the alloy-support synergistic effects for the CO 2 hydrogenation towards methanol on oxide-supported Ni 5 Ga 3 catalysts: An experimental and DFT study (2021) https://doi.org/10.1016/j.apcatb.2021.120842

[19] Hejral, U., Timoshenko, J., Kordus, D., Lopez Luna, M., Divins, N.J., Widrinna, S., Zegkinoglou, I., Pielsticker, L., Mistry, H., Boscoboinik, J.A., Kuehl, S., Roldan Cuenya, B.: Tracking the phase changes in micelle-based NiGa nanocatalysts for methanol synthesis under activation and working conditions. Journal of

Catalysis **405**, 183–198 (2022) https://doi.org/10.1016/j.jcat.2021.11.024

[20] Lin, K.S., Hussain, A., Lin, Y.S., Hsieh, Y.C., Chiang, C.L.: Direct synthesis of CH3OH from CO2 hydrogenation over Ni5Ga3/SiO2 catalysts. Fuel **348**, 128504 (2023) https://doi.org/10.1016/J.FUEL.2023.128504

[21] Zimmerli, N.K., Rochlitz, L., Checchia, S., Müller, C.R., Copéret, C., Abdala, P.M.: Structure and Role of a Ga-Promoter in Ni-Based Catalysts for the Selective Hydrogenation of CO2 to Methanol. JACS Au **4**(1), 237–252 (2024) https://doi.org/10.1021/jacsau.3c00677

[22] Proaño, L., Jones, C.W.: CO2 hydrogenation to methanol over ceria-zirconia NiGa alloy catalysts. Applied Catalysis A: General **669**, 119485 (2024) https://doi.org/10.1016/J.APCATA.2023.119485

[23] Rasteiro, L.F., Assaf, J.M., Assaf, E.M.: Investigation of intermediates formation in the CO2 hydrogenation to methanol reaction over Ni5Ga3-ZrO2-SBA-15 materials prepared via ZrO2 atomic layer deposition. Applied Surface Science **654**, 159444 (2024) https://doi.org/10.1016/J.APSUSC.2024.159444

[24] Zhou, H., Zhang, S., Shao, Y., Liu, S., Fan, X., Chen, H.: Relationship between Structural Properties of the Unsupported Ni5Ga3 Catalyst and Methanol Synthesis Activity. Industrial and Engineering Chemistry Research **63**, 6984 (2024) https://doi.org/10.1021/acs.iecr.4c00241

[25] Jain, A., Ong, S.P., Hautier, G., Chen, W., Richards, W.D., Dacek, S., Cholia, S., Gunter, D., Skinner, D., Ceder, G., Persson, K.A.: Commentary: The materials project: A materials genome approach to accelerating materials innovation. APL Materials **1**(1), 11002 (2013) https://doi.org/10.1063/1.4812323

[26] Tankard, R.E., Romeggio, F., Akazawa, S.K., Krabbe, A., Sloth, O.F., Secher, N.M., Colding-Fagerholt, S., Helveg, S., Palmer, R., Damsgaard, C.D., Kibsgaard, J., Chorkendorff, I.: Stable mass-selected AuTiOx nanoparticles for CO oxidation. Physical Chemistry Chemical Physics (2024) https://doi.org/10.1039/D4CP00211C

[27] Henriksen, T.R., Olsen, J.L., Vesborg, P., Chorkendorff, I., Hansen, O.: Highly sensitive silicon microreactor for catalyst testing. Review of Scientific Instruments **80**(12) (2009) https://doi.org/10.1063/1.3270191

[28] Vesborg, P.C.K., Olsen, J.L., Henriksen, T.R., Chorkendorff, I.B., Hansen, O.: Note: Anodic bonding with cooling of heat-sensitive areas. Review of Scientific Instruments **81**(1), 016111 (2010) https://doi.org/10.1063/1.3277117

[29] Tanuma, S., Powell, C.J., Penn, D.R.: Calculations of electron inelastic mean free paths. V. Data for 14 organic compounds over the 50–2000 eV range. Surface and Interface Analysis **21**(3), 165–176 (1994) https://doi.org/10.1002/SIA.740210302

[30] Hochfilzer, D., Sørensen, J.E., Clark, E.L., Scott, S.B., Chorkendorff, I., Kibsgaard, J.: The Importance of Potential Control for Accurate Studies of Electrochemical CO Reduction. ACS Energy Letters **6**(5), 1879–1885 (2021) https://doi.org/10.1021/ACSENERGYLETT.1C00496/ASSET/IMAGES/LARGE/NZ1C00496{_}0004.J
